# Mono-specific algal diets shape microbial networking in the gut of the sea urchin *Tripneustes gratilla elatensis*

**DOI:** 10.1186/s42523-021-00140-1

**Published:** 2021-11-15

**Authors:** Matan Masasa, Ariel Kushmaro, Esti Kramarsky-Winter, Muki Shpigel, Roy Barkan, Alex Golberg, Abraham Kribus, Nadav Shashar, Lior Guttman

**Affiliations:** 1 Marine Biology and Biotechnology Program, Department of Life Sciences, Ben-Gurion University of the Negev, Eilat Campus, Eilat, Israel; 2 Israel Oceanographic and Limnological Research, The National Center for Mariculture, P.O. Box 1212, 8811201 Eilat, Israel; 3 Avram and Stella Goldstein-Goren, Department of Biotechnology Engineering, Ben-Gurion University of the Negev, P.O.B. 653, 8410501 Beer-Sheva, Israel; 4 Morris Kahn Marine Research Station, The Leon H. Charney School of Marine Sciences, University of Haifa, 3498838 Haifa, Israel; 5 Department of Environmental Studies, Tel Aviv University, P.O. Box 39040, 6997801 Tel Aviv, Israel; 6 School of Mechanical Engineering, Tel Aviv University, P.O. Box 39040, 6997801 Tel Aviv, Israel

## Abstract

**Background:**

Algivorous sea urchins can obtain energy from a diet of a single algal species, which may result in consequent changes in their gut microbe assemblies and association networks.

**Methods:**

To ascertain whether such changes are led by specific microbes or limited to a specific region in the gut, we compared the microbial assembly in the three major gut regions of the sea urchin *Tripneustes gratilla elatensis* when fed a mono-specific algal diet of either *Ulva fasciata* or *Gracilaria conferta*, or an algal-free diet. DNA extracts from 5 to 7 individuals from each diet treatment were used for Illumina MiSeq based 16S rRNA gene sequencing (V3–V4 region). Niche breadth of each microbe in the assembly was calculated for identification of core, generalist, specialist, or unique microbes. Network analyzers were used to measure the connectivity of the entire assembly and of each of the microbes within it and whether it altered with a given diet or gut region. Lastly, the predicted metabolic functions of key microbes in the gut were analyzed to evaluate their potential contribution to decomposition of dietary algal polysaccharides.

**Results:**

Sea urchins fed with *U. fasciata* grew faster and their gut microbiome network was rich in bacterial associations (edges) and networking clusters. Bacteroidetes was the keystone microbe phylum in the gut, with core, generalist, and specialist representatives. A few microbes of this phylum were central hub nodes that maintained community connectivity, while others were driver microbes that led the rewiring of the assembly network based on diet type through changes in their associations and centrality. Niche breadth agreed with microbes' richness in genes for carbohydrate active enzymes and correlated Bacteroidetes specialists to decomposition of specific polysaccharides in the algal diets.

**Conclusions:**

The dense and well-connected microbial network in the gut of Ulva-fed sea urchins, together with animal's rapid growth, may suggest that this alga was most nutritious among the experimental diets. Our findings expand the knowledge on the gut microbial assembly in *T. gratilla elatensis* and strengthen the correlation between microbes’ generalism or specialism in terms of occurrence in different niches and their metabolic arsenal which may aid host nutrition.

**Supplementary Information:**

The online version contains supplementary material available at 10.1186/s42523-021-00140-1.

## Background

The ability of microbes to colonize different niches, i.e., their niche breadth, determines the composition and therefore networking of the microbial assembly when challenged by various environmental forces [1]. Generalist microbes are capable of occupying various niches, while specialists are those with a narrower habitat, i.e., found in fewer niches. Specialization can also be determined as unique if the microbe appears consistently only in a particular niche, condition or occasion [2, 3]. With the constant development of new tools for analyzing microbial communities, specific platforms for network analyses have also emerged. These tools allow the discrimination of association networks following their topologies as to density, clustering rate, diameter, length of shortest path, and other indices [4]. Accordingly, the organizational level of the community can be highly random, small-world, or scale-free [4, 5]. In random networks, the connectivity of microbe nodes is not easily disrupted since many microbial nodes can be reached by any other node through a short number of steps, and the associating nodes are more likely to be neighbors of each other [6]. A closer investigation into the different nodes in an assembly enables us to reveal the key hub microbes with the highest centrality and connectivity, as demonstrated by a fair number of edges (associations) and their proximity to other microbe nodes [7]. Likewise, driver microbes with the greatest contribution to the rewiring of a network under a certain weight (i.e., niche) can be identified by the changes in their associating members, type of association, and centrality [8].

Among numerous environmental forces, diet is considered to influence the composition of the gut-microbe assembly (GMA) of various aquatic invertebrates [9]. A particularly strong effect may be attained if animals are restricted to only one particular nutrient source. A prime example is sea urchins that can gain their energy from a single, usually preferred, algal species [10, 11]. In many cases such preference has been credited with positive impacts on performance, e.g., growth, gonad color and somatic index [12, 13].

Evidence that sea urchins that can be cultured when fed different macroalgae, each as a sole energy source, are favorable for study of the responses of GMA and microbial networking to changes in diet. Toward this goal we selected the sea urchin *Tripneustes gratilla elatensis* as a model algivorous species to examine whether and how diet restriction to a single species of alga, either *Ulva fasciata* (Chlorophyta) or *Gracilaria conferta* (Rhodophyta), will affect the gut microbial assembly compared to a plant-rich but algal-depleted pelleted diet. Not only changes in the composition and networking that could occur at the level of the entire assembly were considered, but also specific changes in the associations and centrality in the network of specific microbes in the assembly. It was further hypothesized that such changes may be more prominent in one of the three major regions in the gut (esophagus, stomach, or intestine) rather than over the entire organ. Hence, the experiment aimed at identification of the key microbes or microbe taxa in the gut of *T. gratilla elatensis* through their generalism or specialism in the different niches examined (diet types and gut regions) as per their occurrence in broader or narrower niches and also their content of genes for carbohydrates decomposition as potential contribution in feed metabolism.

## Methods

### Sea urchin cultivation with experimental diets

Fifty-four 6-month-old individuals of *Tripneustes gratilla elatensis* from a single hatch at the National Center for Mariculture (NCM, Eilat, Israel), were assigned for the feeding trial in an in-house experimental system which allows control of environmental conditions including the recommended ambient light for studies of this species [14]. This system comprises nine 90L rectangular tanks and allows maintenance of water quality, temperature, and oxygen saturation via a rapid flow of fresh filtered sea water, as well as continuous aeration of the tanks. Prior to restricting the diet to a given experimental feed treatment, all sea urchins were fed the algal diet of *Ulva fasciata* together with *Gracilaria conferta* recommended at the local integrated multi-trophic aquaculture system (IMTA)*.* After two weeks of acclimation, homogeny in the physical parameters of body (‘test’) diameter (85.2 ± 2 mm) and weight (272.4 ± 17 g wet weight) between individuals was validated.

The experimental diets were comprised solely of either fresh* Ulva fasciata* (‘*Ulva* diet’) or fresh *Gracilaria conferta* (‘*Gracilaria* diet’) grown in the local IMTA system, or a pelleted feed made of plant-meals that lack algae or any marine footprint but contain whole wheat, rapeseed, soya pulp, corn starch and other essential minerals and vitamins (Additional file 1: Table S1)*.* These diets differ greatly in their polysaccharide content. In *Gracilaria,* galactans are the primary polysaccharides, classified as agarans and carrageenans, and the latter also consist of sulfate ester groups in their repeating disaccharides in the poly-chain [15]. In *Ulva,* sulfated ulvans are the main cell-wall polysaccharides and their content in dry biomass may reach 38–54% [16]. In contrast to these marine polysaccharides in the algal diets, the primary polysaccharides in the pelleted feed used are plant cellulases and starch. The feeding trial was set in a completely randomized block design with triplicate tanks for each treatment containing nine individuals each, all of which were nourished ad libitum for a period of eight weeks. Feeding ended forty-eight hours prior to examination of the sea urchins in order to minimize remnant digesta in the gut. The sea urchins’ initial and final diameter, weight (dry and wet weight), and gonad weight, were measured in three randomly selected individuals in each tank. The specific growth rate and gonadosomatic index were then calculated as described elsewhere [12].

### DNA extraction and sequencing

Three sea urchins from each tank (nine per diet type) were sacrificed for DNA sampling from the different gut regions. The tubular digestive tract was removed and separated into its three major regions [17] of the esophagus, which is closest to the mouth and where ingested food is covered by a mucous layer [18], and the anterior stomach and posterior intestine where secretion of digestive enzymes and nutrient absorption take place, respectively [19, 20]. Dissection was performed using sterile instruments, while residual digesta was removed, and the remaining tissue was rinsed gently with Ultra-Pure water (Additional file 1: Fig. S1a-d). Gut samples were homogenized in FastPrep-24™ 5G homogenizer (MP Biomedicals) and gDNA was extracted using the PureLink™ Microbiome DNA Purification Kit (Thermo Fisher Scientific, USA) following the manufacturer's protocol. Only DNA of high quality and quantity was amplified with the 515F (GTGYCAGCMGCCGCGGTAA) and 926R (CCGYCAATTYMTTTRAGTTT) primer set [21] by denaturation at 95 °C for 5 min, 28 cycles at 94 °C for 45 s, 50 °C for 60 s, 72 °C for 90 s, and a final elongation at 72 °C for 10 min. Bacterial 16S rRNA gene sequencing was performed at the Research Resources Center (University of Illinois, Chicago) by the Illumina MiSeq platform [22] using the reagent kit v3. Quality and quantity assurance included examination of negative and positive control samples (using fecal samples as positive control) on which all procedures both before and after sequencing were performed.

### Data analysis

Analyses of GMA were performed on 15, 18, and 21 samples from urchins fed with diets of *Ulva,* algae-free pellets*,* and *Gracilaria*, respectively. Different gut regions were represented equally in each sample set. Bioinformatics analysis was performed using the Quantitative Insights into Microbial Ecology pipeline (QIIME; version1.9.1) [23]. After removing primers and linkers, pair-end reads were merged using PEAR [24], and sequences with a quality score below 30 or shorter than 300 bp were filtered out. Sequences were clustered into operational taxonomic units (OTUs) at 97% similarity cutoff using UCLUST [25] and the Silva database (ver. 132) [26], and chimera sequences were removed by ChimeraSlayer [27]. A subsequent alignment step by PyNAST [28] identified master sequences according to the most abundant criteria. Taxonomy assignment was established using the UCLUST classifier while discarding OTUs represented by less than five sequences or those identified as chloroplast or mitochondria. A Linear discriminant analysis Effect Size (LEfSe) [29] at false discovery rate (FDR) *P* < 0.01 was performed to identify bacterial taxa that can be considered as biomarkers of a certain diet or gut region.

Each of the microbes in the assembly was examined separately to determine whether it falls under the definition of core or unique microbe and secondly as an either generalist or specialist microbe. Core microbes were identified as those that appeared in at least 85% of samples of any specific habitat within the examined variable of diet, gut region, or both. On the other end, unique microbes were those that appeared in at least 85% of the samples of a specific gut region or diet but in no more than 25% of the samples of each of the other two diets or gut regions. Following this classification, niche breadth of each of the microbes was measured as recommended [30] by ranking OTU evenness of occurrence across various habitats (Shannon–Weaver diversity) [31], occupancy across habitats (Richness) [32], and the Levin's index [33]. Microbes with an overall rank of niche breadth within the top or bottom decile were defined as generalists or specialists, respectively, while all other microbes were defined as non-significant. Microbial association networks were analyzed using MetagenoNets [34] and the latent variable model Correlation interference for Compositional data through Lasso (CCLasso) [35] after log ratio transformation and measurement of edge weight (significant level of *P* < 0.005 and permutation of 200) for each node pair, excluding OTUs with low prevalence (< 0.001%) and occurrence (< 10%). Topology indices of density, diameter, cluster coefficient, average path length, and Jaccard edge index were analyzed for networks and participating nodes by Cytoscape [36]. The randomization level of association networks was evaluated following the common distribution model of nodes’ number of degrees [37]. Following the model, a random network will reveal Poisson distribution with many of the nodes having about the same number of degrees, while in a scale-free network a power-tail distribution is formed by a high number of nodes that have few degrees and vice versa. Hub nodes were identified using CentiServer [38] following their high degrees (edges), proximity centrality (short distance to any other node), between-ness (fraction of cases when node is in-between shortest track of all other pair nodes) and the Kleinberg's hub centrality [39] score as recommended [7, 40]. In addition to hub taxa, driver nodes are defined as those leading to rewiring of the association network under a specific treatment. Therefore, they present a low proportion of shared edges with other networks (Jaccard edge index; JEI), as well as exclusive enrichment of interacting partners (neighbor shift score; NESH) and a greater centrality (i.e., greater occurrence frequency in paths of other nodes) as compared to other nets (∆B). Such nodes were extracted from data using NetShift [8] employing a combined threshold of JEI < 0.3, NESH > 1.8, and a non-zero ∆B which was validated via pair-wised tests against each of the other two networks (diets or gut regions). Since identification was carried out in sets of paired networks, it should be noted that driver nodes identified here achieved the driver criteria in one specific niche network when compared in pair to each of the other two networks. Moreover, such microbes failed the criteria when compared with the other two network pairs.

A further examination of the predicted metabolic and functional roles of the key bacterial taxa identified in *T. gratilla elatensis* was performed. To do this, the sequences of OTUs that were identified as key hub microbes and those categorized as core, core-generalists, generalists, specialists, or unique in a specific niche, were uploaded to the NCBI database for blasting with megablast [41]. Bacterial strains with the highest number of hits and an available representative genome were selected for further analysis of their metabolic repertoire. Considering the differences in the biochemical content of the experimental diets, the genomes of these bacteria were analyzed in the Carbohydrate-Active enZYmes database (CAZy) using the dbCAN2 meta server [42] to identify annotated genes that encode carbohydrate-active enzymes (CAZymes) of carbohydrate binding, carbohydrate esterases, glycoside hydrolases, and glycoside transferases. A heat-map of these results was produced to visualize the variation in the content and number of copies of the glycoside hydrolase (GH) and polysaccharide lyase (PL) genes (enzyme classes EC 3.2.1.-, and 4.2.2.-, respectively) between the selected microbes.

### Statistical analyses

MicrobiomeAnalyst [43, 44] was used for examining each diet or gut region separately and also the combined effects of both these variables together. Rarefaction of data at a sequencing depth of 10,000 reads resulted in 51 samples for which differences in ecological indices of richness, alpha and beta diversity were measured by Kruskal–Wallis and post hoc Dunn’s multiple comparison test. Differences in microbial community composition were measured by Bray–Curtis dissimilarity and Permutational multivariate analysis of variance (PERMANOVA). Differences in relative abundance were verified by Kruskal–Wallis, at a false discovery rate (FDR) of *P* < 0.01. We selected the non-parametric Kruskal–Wallis test after refuting the null hypothesis and confirming that data in the sequencing results are not normally distributed following a Shapiro–Wilk test of distribution of the residuals (*P* < 0.0001).

## Results and discussion

### Diet shapes the gut microbe assembly of T. gratilla elatensis

No mortality or morbidity of sea urchins was observed during the entire period of the feeding trial. Sea urchin individuals fed the *Ulva* diet had a growth rate of 0.33% d^−1^ as compared to 0.27 or 0.1% d^−1^ on diets of *Graciaria* or pellets (*P* < 0.05), respectively (Additional file 1: Table S2). The faster growth rate under dietary *Ulva* as compared to *Gracilaria* or pellet diet is consistent with a previous study of *T. gratila elatensis* when fed similar diets of *Ulva, Gracilaria* and pellets over a much longer culture period of 400 days [12].

**Fig. 1:**
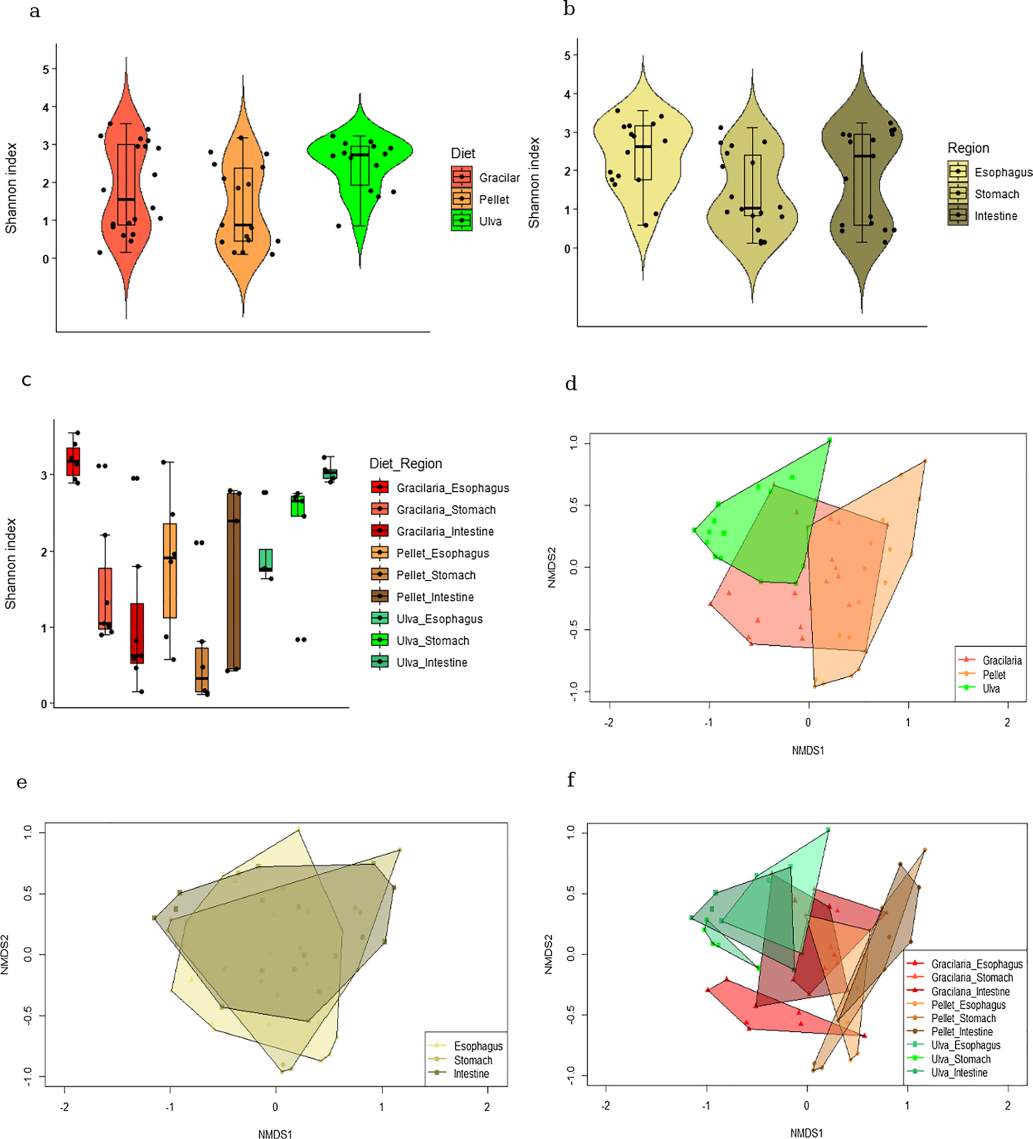
Richness, diversity and similarity of *T. gratilla elatensis* GMA under different variables of diet and gut region. Shannon index considering **a** different diets, **b** gut regions, or **c** both variables (n = 51). Box plots represent a level of 95% confidence interval, bar plots represent the standard error, straight lines represent the median, and the black dot represents the mean. Dissimilarity between GMA is demonstrated by non-metric multidimensional scaling (NMDS) based on Bray–Curtis dissimilarities between GMA in different **d** diets **e** gut regions, or **f** both forces (n = 51)

The fifty-one samples that reached sequencing depth of 10,000 reads resulted in 988,513 high-quality reads (18,305 ± 5208 per sample) that were clustered into 434 unique OTUs (117 ± 48 per sample; Additional file 1: Table S3). Neither diet nor gut region alone affected the richness of the GMA (H_50,9_ = 15.37; *P* > 0.05). The richest assembly was identified in the esophagus of the *Gracilaria-*fed sea urchins and the poorest assembly was found in the stomach of the pellet-fed sea urchins (Additional file 1: Fig. S2). The more diverse community in the anterior esophagus (H_50,3_ = 6.483; *P* < 0.05; Fig. 1a, b) agrees with results reported in previous studies on the sea urchin *Lytechinus variegatus* either prior to or after capture and rearing on artificial diet [45, 46]. This may be attributed to the rapid interactions with the external water environment and short retention time of ingested feed in the esophagus [47]. Lower pH and oxygen and high secretion of digestive enzymes in the stomach [19, 48] may have limited the assembly diversity in this niche. A similar characteristic was noted in those fed with artificial pellets (Fig. 1c), perhaps indicating disruption of the assembly due to the lack of algae or any other marine footprint in this diet. Diet type restriction induced dissimilarity between gut assemblies (F_50,3_ = 8.2, *P* < 0.01, R^2^ = 0.25, Stress = 0.143; Fig. 1d, e). The effect was intense enough to also differentiate assemblies of the different gut regions (F_50,9_ = 6.16, *P* < 0.01, R^2^ = 0.539; Fig. 1f; Additional file 1: Fig. S3a-c), with the strongest impact seen in the *Gracilaria*-fed sea urchins (*P* < 0.001 Additional file 1: Fig. S3a). This combined effect of the diet and gut region variables together (as compared to the effect of only diet or only gut region) resulted in a more diverse assembly in the anterior esophagus as compared to posterior regions in *Gracilaria-*fed urchins (*P* < 0.01*),* while an exact opposite pattern was noted in *Ulva-*fed sea urchins (*P* < 0.02*)* (Fig. 1c). An interesting finding was the relatively low variation in Shannon index of diversity within samples from different sea urchins when fed the *Ulva* diet, in each of the examined gut regions of these sea urchins (Fig. 1c). This can be attributed to the relatively consistent prevalence of the dominant taxa in the *Ulva-* fed sea urchins across samples of different biological replicates (sea urchin individuals; Additional file 1: Fig. S4b), which also resulted in a relatively low variation within individuals in terms of the community composition and diversity.

### Predominance of bacterial taxa in the gut of T. gratilla elatensis

**Fig. 2:**
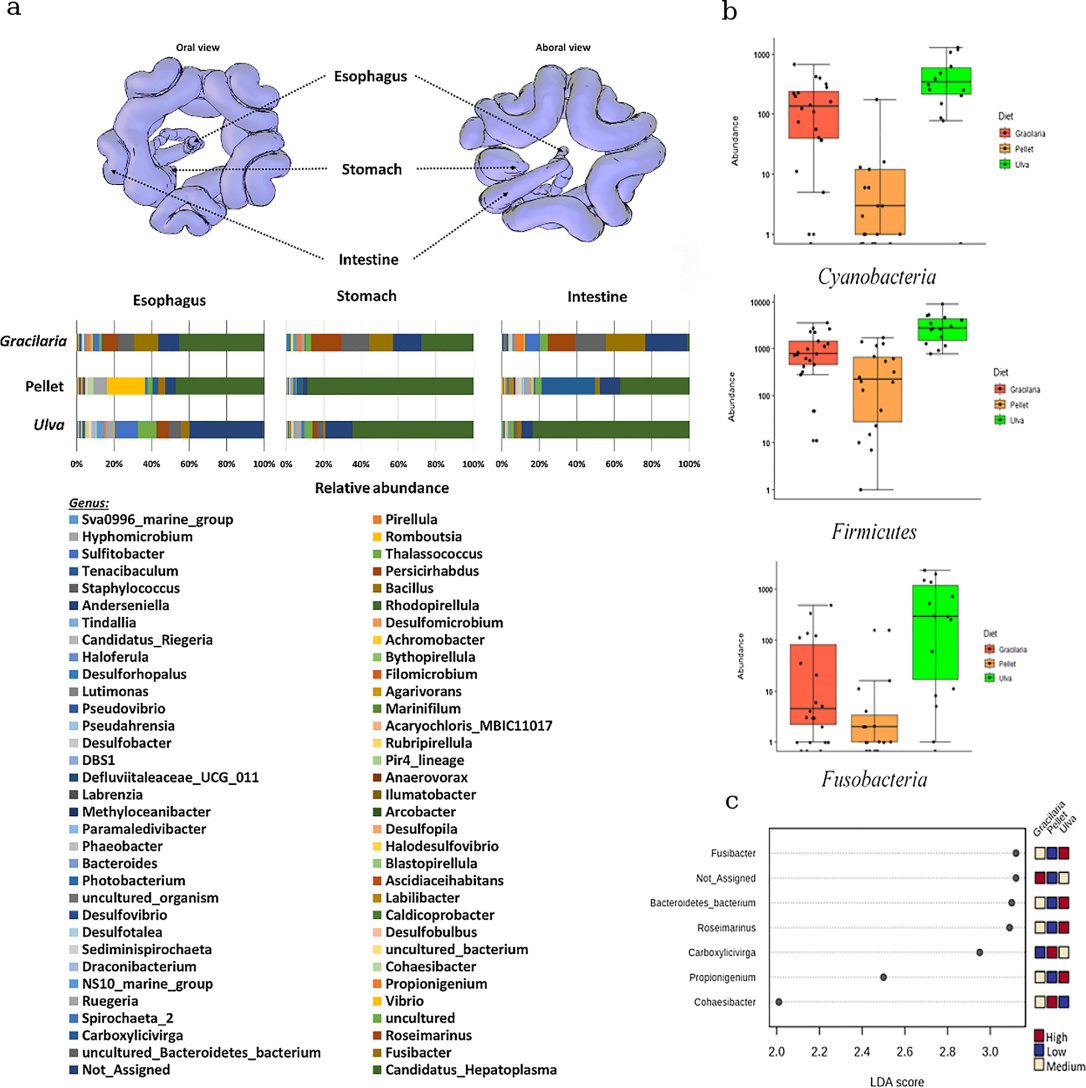
*T. gratilla elatensis* GMA composition under different variables of diet and gut region. **a** Illustration of sea urchin digestive tract (performed using magnetic resonance imaging [49] and image illustrator at https://www.nhm.ac.uk/our-science/data/echinoid-directory) followed by the relative abundance of *T. gratilla elatensis* gut microbes (Genera) in different gut regions and under each diet (n = 54). **b** Log-transformed count of bacterial phyla with greatest differences in prevalence under a particular diet, middle line represents median, and whiskers are drawn from the 10th to 90th percentiles (n = 54). **c** Gut bacterial genera identified as diet-biomarkers in *T. gratilla elatensis* via Linear discriminant analysis (n = 54)

None of the 110 genera (Fig. 2a) from 17 bacterial phyla (Additional file 1: Fig. S4a, Table S4) predominated in a particular gut region, but the three taxa, *Cyanobacteria*, *Firmicutes,* and *Fusobacteria,* were predominant in the assembly in *Ulva*-fed sea urchins (Fig. 2b, *P* < 0.001) and exhibited (each in itself) a relatively similar prevalence in the different gut regions in all the examined diets. An opposite prevalence pattern of *Spirochaetes* vs. *Tenericutes* was evident under the *Gracilaria* diet. The *Spirochaetes*, primarily represented by the genus *Spirochaeta,* predominated in the esophagus and decreased in posterior regions, while the *Tenericutes*, which was primarily represented by a single OTU assigned as genus *Candidatus hepatoplasma*, predominated in the intestine and stomach and was lower in the esophagus (*P* < 0.001; Additional file 1: Fig. S4b). Fluctuations in prevalence allowed identification of potential diet biomarkers in the GMA (Fig. 2c) as the genera *Fusibacter*, uncultured Bacteroidetes bacterium, *Roseimarinus*, and *Propionigenium* for *Ulva; Carboxylicivirga* and *Cohaesibacter* for pelleted feed; and unassigned for *Gracilaria;* but none were specific to any gut region.

### Niche specification in the gut microbial assembly of sea urchins

**Fig. 3:**
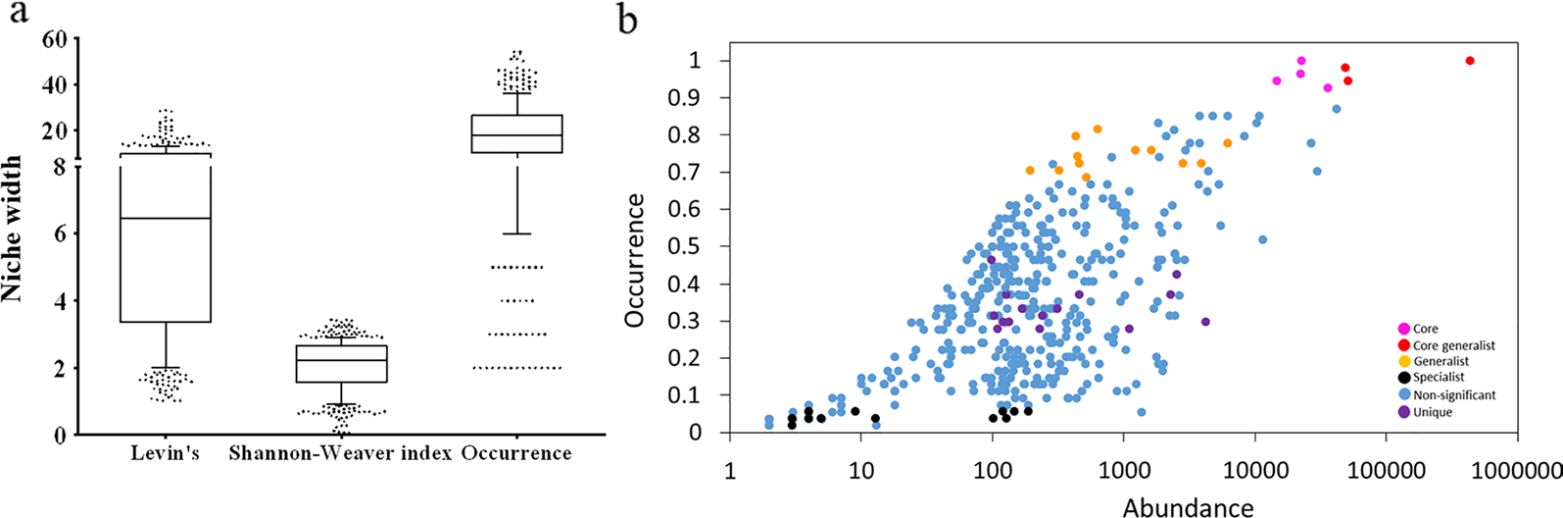
**a** Niche breadth of microbes in sea urchin GMA following the different measured indices of Levin's, Shannon–Weaver, and occurrence. Middle line in box plots represents mean value and whiskers are drawn from the 10th to 90th percentiles. Generalist or specialist microbes are shown at top or lowest decile, respectively (n = 434). **b** Occurrence and cumulative abundance of each of the microbes in GMA indicates differences of niche breadth and specialization in unique habitats. Each dot represents one microbe with a color indication indicating core, core-generalist, generalist, specialist, unique, or not significant (n = 434)

Generalist and core microbes colonize the majority of examined ecological niches, i.e., the different gut regions of *T. gratilla elatensis,* fed on the different diets. Therefore, these microbes may be highly important in aiding their host in changing environments [50, 51]. Adhering to the fixed rule of colonization of at least 85% of the examined samples (across any gut region and/or diet), the core microbes identified in the gut of *T. gratilla elatensis* included five OTUs belonging to taxa *Candidatus Hepatoplasma*, *Ruegeria*, *Vibrio*, and two uncultured members of the order *Bacterioidia* (uncultured bacterium and uncultured Bacteroidetes bacterium) (Table 1). Two additional taxa of uncultured *Fusibacter* and *Roseimarinus* were thusly determined as core microbes in all the examined gut regions and under diets of *Ulva* and *Gracilaria*. However, they marginally failed the selection threshold criteria in the pellet-fed urchins, appearing in only 83% of the samples under this diet. The cumulative abundance of each of the identified core microbes was above 10,000 reads (Fig. 3a). Moreover, the relative abundance of a core microbe obtained in any sample was greater than 0.01%. These findings on the occurrence and abundance of core microbes may aid in their confirmation as resident microbes in the gut of *T. gratilla elatensis* rather than transient microbes which may only pass through this organ.

**Table 1:** Bacterial characterization following niche breadth and occurrence in the different examined niches differentiates the assembly microbes into core, core generalists, generalists, specialists and unique microbes

Definition	OTU ID	Phylum	Class	Order	Family	Genus	Species	Levin's	Shannon–Weaver index	Occurrence
Core	denovo32222	Bacteroidetes	Bacteroidia	Bacteroidales	Prolixibacteraceae	Roseimarinus	Uncultured Bacteroidetes bacterium	10.47	2.68	0.93
	denovo7299	Bacteroidetes	Bacteroidia	Bacteroidetes VC2.1 Bac22	Uncultured bacterium			10.08	2.79	0.94
	denovo426	Proteobacteria	Alphaproteobacteria	Rhodobacterales	Rhodobacteraceae	Ruegeria		12.51	2.94	1.00
	denovo12912	Proteobacteria	Gammaproteobacteria	Vibrionales	Vibrionaceae	Vibrio		3.00	1.73	0.96
Core generalist	denovo7539	Bacteroidetes	Bacteroidia	Bacteroidales	vadinHA21	Uncultured Bacteroidetes bacterium		17.39	3.07	0.98
	denovo3738	Firmicutes	Clostridia	Clostridiales	Family XII	Fusibacter	Uncultured Fusibacter sp.	13.87	2.95	0.94
	denovo31035	Tenericutes	Mollicutes	Entomoplasmatales	Entomoplasmatales Incertae Sedis	Candidatus Hepatoplasma		28.63	3.44	1.00
Generalist	denovo34858	Unassigned						28.37	3.43	0.76
	denovo2269	Unassigned						28.26	3.42	0.69
	denovo36881	Proteobacteria	Alphaproteobacteria	Rhodobacterales	Rhodobacteraceae			23.52	3.41	0.80
	denovo582	Unassigned						20.40	3.24	0.72
	denovo20261	Unassigned						20.12	3.14	0.54
	denovo29824	Unassigned						19.99	3.23	0.67
	denovo34968	Firmicutes	Clostridia	Clostridiales	Caldicoprobacteraceae	Caldicoprobacter	Uncultured bacterium	19.78	3.21	0.72
	denovo2328	Proteobacteria	Gammaproteobacteria	Betaproteobacteriales	Burkholderiaceae	Achromobacter	Achromobacter xylosoxidans subsp. xylosoxidans	18.60	3.26	0.70
	denovo23618	Proteobacteria	Gammaproteobacteria	Vibrionales	Vibrionaceae	Photobacterium	Uncultured gamma proteobacterium	17.71	3.09	0.76
	denovo29071	Cyanobacteria	Melainabacteria	Gastranaerophilales				16.70	3.10	0.78
	denovo20151	Planctomycetes	Planctomycetacia	Pirellulales	Pirellulaceae	Blastopirellula		14.78	3.07	0.74
	denovo33543	Planctomycetes	Planctomycetacia	Planctomycetales	Rubinisphaeraceae	Uncultured	Uncultured bacterium	14.69	3.07	0.70
	denovo18322	Proteobacteria	Alphaproteobacteria	Rhizobiales	Methyloligellaceae	Methyloceanibacter	Uncultured bacterium	14.55	3.06	0.81
	denovo36317	Proteobacteria	Deltaproteobacteria	Desulfobacterales	Desulfobulbaceae	Desulfotalea		13.89	3.00	0.72
	denovo11613	Planctomycetes	Planctomycetacia	Pirellulales	Pirellulaceae	Rubripirellula	Uncultured bacterium	13.38	3.00	0.72
Specialist	denovo2310	Bacteroidetes	Bacteroidia	Flavobacteriales	Flavobacteriaceae			1.60	0.56	0.04
	denovo43468	Bacteroidetes	Bacteroidia	Cytophagales	Cyclobacteriaceae	Reichenbachiella		1.60	0.56	0.04
	denovo13979	Bacteroidetes	Bacteroidia	Cytophagales	Cyclobacteriaceae	Fabibacter	Uncultured bacterium	1.59	0.68	0.06
	denovo32773	Unassigned						1.51	0.62	0.06
	denovo1069	Thaumarchaeota (Archaea)	Nitrososphaeria	Nitrosopumilales	Nitrosopumilaceae	Candidatus Nitrosopumilus	Uncultured archaeon	1.47	0.50	0.04
	denovo9503	Bacteroidetes	Bacteroidia	Flavobacteriales	Cryomorphaceae	Uncultured	Uncultured bacterium	1.17	0.27	0.04
	denovo10645	Bacteroidetes	Bacteroidia	Sphingobacteriales	Lentimicrobiaceae	Uncultured bacterium		1.04	0.11	0.06
	denovo3573	Firmicutes	Negativicutes	Selenomonadales	Veillonellaceae	Veillonella		1.02	0.06	0.04
	denovo6928	Proteobacteria	Alphaproteobacteria	Rhizobiales	Rhizobiaceae	Phyllobacterium		1.02	0.05	0.04
	denovo38262	Bacteroidetes	Bacteroidia	Flavobacteriales	Flavobacteriaceae	Maritimimonas		1.92	0.67	0.04
	denovo5098	Bacteroidetes	Bacteroidia	Sphingobacteriales	Lentimicrobiaceae	Uncultured bacterium		1.81	0.69	0.04
	denovo9355	Proteobacteria	Deltaproteobacteria	Desulfobacterales	Desulfobacteraceae	Desulfobacter		1.80	0.64	0.04
Ulva unique	denovo10328	Bacteroidetes	Bacteroidia	Bacteroidales	Prolixibacteraceae	Roseimarinus		7.68	2.23	0.37
	denovo43431	Bacteroidetes	Bacteroidia	Bacteroidales	Prolixibacteraceae	Roseimarinus		7.91	2.27	0.28
	denovo9352	Bacteroidetes	Bacteroidia	Bacteroidales	Prolixibacteraceae	Roseimarinus		9.43	2.43	0.33
	denovo4973	Bacteroidetes	Bacteroidia	Bacteroidales	Prolixibacteraceae	Roseimarinus		9.25	2.37	0.28
	denovo36133	Bacteroidetes	Bacteroidia	Bacteroidales	vadinHA21	Uncultured Bacteroidetes bacterium		10.81	2.57	0.31
	denovo6275	Cyanobacteria	Melainabacteria	Gastranaerophilales				6.55	2.27	0.37
	denovo25524	Firmicutes	Clostridia	Clostridiales	Defluviitaleaceae	Defluviitaleaceae UCG-011	Uncultured bacterium	11.45	2.64	0.37
	denovo19840	Spirochaetes	Spirochaetia	Spirochaetales	Spirochaetaceae	Spirochaeta 2	Spirochaeta isovalerica	6.81	2.16	0.30
	denovo23305	Spirochaetes	V2072-189E03	Uncultured organism				3.59	1.69	0.30
	denovo11880	Tenericutes	Mollicutes	Mollicutes RF39	Uncultured bacterium			5.28	2.11	0.33
	denovo30083	Unassigned						5.82	2.00	0.28
	denovo12333	Unassigned						7.55	2.35	0.31
	denovo10114	Unassigned						8.81	2.42	0.30
Gracilaria unique	denovo26795	Planctomycetes	vadinHA49	Uncultured bacterium				14.38	2.88	0.46
	denovo796	Bacteroidetes	Bacteroidia	Cytophagales	Cyclobacteriaceae			6.47	2.21	0.43
Ulva and Gracilaria unique	denovo43602	Unassigned						7.49	2.62	0.67

Indices of niche breadth (Levin's, Shannon–Weaver) and occurrence are provided for each OTU

Identified generalists included 18 microbes (8 of them assigned to phylum *Proteobacteria* or class *Planctomycetacia*) with relatively high occurrence (> 54%), diversity (> 2.95), and niche breadth (> 13.38; Fig. 3a). The 12 identified specialists were mostly annotated to *Bacterioidetes* (Table 1; Fig. 3a). Three core microbes of the respective genera of *C. hepatoplasma,* uncultured Bacteroidetes bacterium*,* and *Fusibacter* were also among generalists (i.e., core-generalists) and revealed the highest cumulative abundance (Fig. 3b). The highest occurrence and abundance of these core-generalists agrees with the predomination of these taxa in the GMA of other sea urchin species as well [52–54]. A recent model highlighted the important role of the generalist-specialist evolutionary cycle which allows the spreading of specialist species across diverse environments, thereby maintaining taxonomic diversity [2] while at the same time influencing the overall community and its differentiation [2, 3]. Following the model, the generalist-specialist cycle includes the expansion of generalist microbes across various ecosystems, where local environmental forces determine the specialization of some of their descendants in the specific niche, hence the latter’s determination as specialist microbes [2]. In another study, core microbes in fish gut revealed high strain variability [30].

Findings in the current study may suggest the fitness of phylum *Bacteroidetes* to this generalists-specialists cycle model with core and core-generalist OTUs (of genus of *Roseimarinus* and uncultured Bacteroidetes bacterium, respectively) as potential founders in the gut assembly of *T. gratilla elatensis*, and several other OTUs of these phyla (four of the genus *Roseimarinus* and one uncultured Bacteroidetes bacterium) identified as specialist microbes that occupied a narrower unique niche of the gut of *Ulva-*fed sea urchins. The fact that *Bacteroidetes* reveal high plasticity and genetic rearrangement, allowing their rapid adaptation to distinct ecological niches [55], makes them more adequately fit the suggested evolutionary model in the examined sea urchin. Despite the significant differences in niche breadth between generalists and unique or specialist microbes, the total abundance of microbes from these groups was relatively similar (Additional file 1: Fig. S5). This indicates that the prevalence of unique or specialist microbes surpassed that of generalists in each of the niches they occupied. This notwithstanding, the core microbes were present in the highest abundance in these niches.

Intriguingly, unique microbes were identified only in sea urchins fed with algae: Thirteen of these in *Ulva-*fed (annotated to phyla *Bacterioidetes, Cyanobacteria, Firmicutes, Spirochetes, Tenericutes*, and unassigned), two in *Gracilaria*-fed (*Bacterioidetes* and *Planctomycetes*), and one unassigned taxon that was shared between these diets (Table 1). One explanation for the *Ulva-* or *Gracilaria-*unique microbes identified here may be the previous identification of members of *Bacteroidetes*, *Cyanobacteria, Firmicutes*, *Spirochaetes*, and *Plancomycetes* taxa as epibionts on these algae [56, 57]. However, the relatively high Shannon diversity and Levin's indices of many of these OTUs that were measured in sea urchins fed these diets may also be explained by high specialization of these microbes in the narrow diet-mediated niches. This is particularly evident in the sea urchin-associated *Bacteroidales* and *Clostridales* fed on an *Ulva* diet and in sea urchin-associated uncultured bacterium (*Planctomycetes*) fed on the *Gracilaria* diet. Correspondingly, the contribution of these microbes to the decomposition of the complex polysaccharides in *Ulva* or *Gracilaria* should be considered, particularly since, despite their algivorous life style, sea urchin digestive systems contain few algal polysaccharide digestive enzymes [47, 58].

### Assembly connectivity is determined via niche-specific hub and driver microbes

**Fig. 4:**
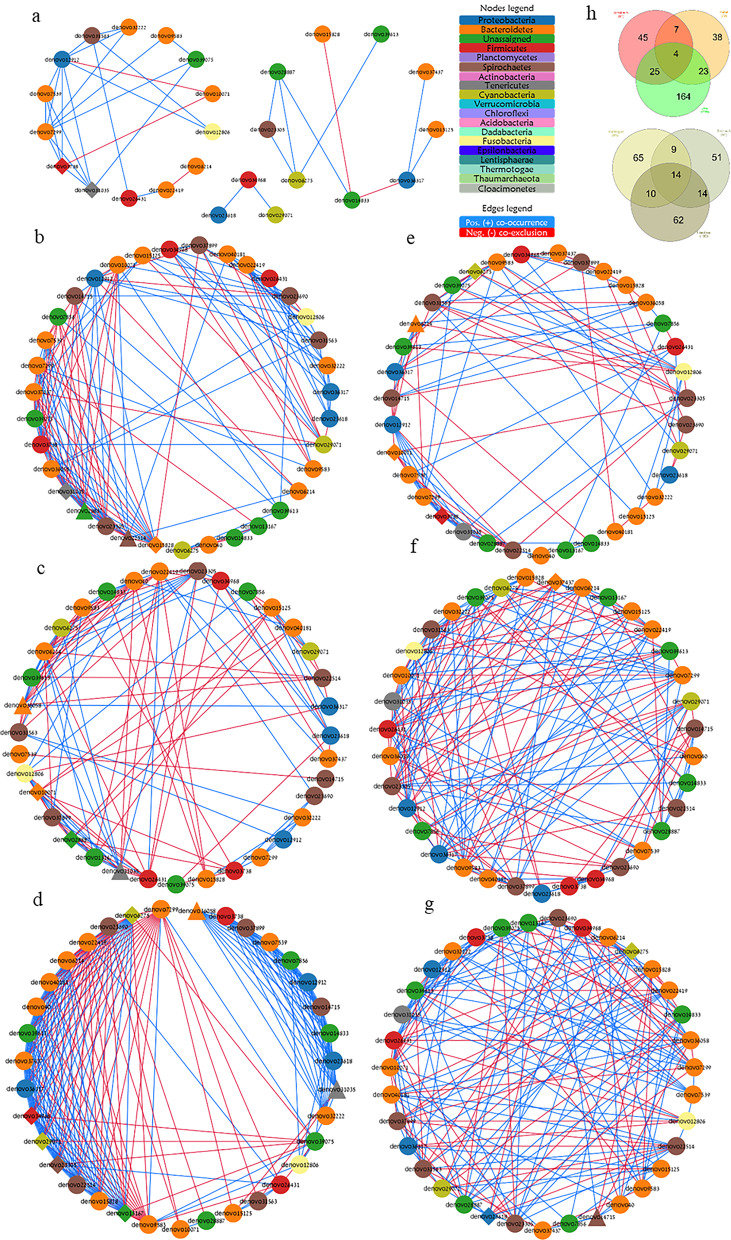
Association network of *T. gratilla elatensis* GMA under different variables of diet and gut region. Association network presenting **a** the general network (regardless of diet or region), and in different examined niches of diets **b** *Gracilaria*, **c** Pellet, or **d** *Ulva*; or gut regions **e** esophagus, **f** stomach, or **g** intestine. Diamonds indicate hub nodes and triangles indicate driver nodes. Nodes are colored following annotation at Phyla level. Edge color indicates type of association as either co-occurrence (blue) or co-exclusion (red). **h** Venn diagrams reveal number of shared or unique associations in networks of different diets or gut regions (n = 54)

The overall association network of the GMA consisted of 27 nodes (each representing a single OTU/microbe) with 37 edges (i.e., of either co-occurrence or co-exclusion; also referred to as degrees). This network, however, was separated topologically and contained three sub-nets, each containing many fewer clusters of the participating microbes (Fig. 4a). This low connectivity of the general network highlights the importance of investigating microbial networks in the narrower niches of each different diet or functional region (Fig. 4b–g) to also allow the identification of niche-specific associations, clusters, hub microbes, or other changes. Examination of the variation in number of degrees between participating nodes in the different networks revealed a Poisson distribution of the degrees per node in networks of the different gut regions but a power-law tail distribution of this index in networks under different diets (Additional file 1: Fig. S6). These results suggest that the microbial networks in different gut regions or under different diets can be differentiated into random or scale-free networks, respectively [59]. Notably, many nodes in the network of the *Ulva-*fed sea urchins possessed a high number of degrees and formed a left tail distribution shape. This was exceptional compared to the other two diets, where only a few nodes revealed a high number of degrees, i.e., right tail distribution (Additional file 1: Fig. S6a-g). Considering the nature of associations, i.e., positive co-occurrence or negative co-exclusion, the number of positive associations in all examined niches surpassed that of the negative ones, with the greatest fraction of the total edges in the *Ulva* and intestine networks. However, the nature of many of the shared associations between networks was of competition/co-exclusion between *Cyanobacteria* and *Bacteroidetes* or *Firmicutes* (diets), or between nodes of *Cyanobacteria, Spirochaetes, Firmicutes, Tenericutes*, and *Bacteroideteds* (gut regions) (Additional file 1: Table S5).

Examination of the topology indices of the co-association networks (Additional file 1: Fig. S7a–g) revealed similarity in terms of the number of edges between participating nodes in networks of the different gut regions (14–16% similarity), but under different diets  the *Ulva* network revealed a greater dissimilarity from any other diet network with *ca* 2.5-fold more edges (Additional file 1: Fig. S7b). The significant high edges number in the *Ulva* network may indicate cross feeding relationships and richness in feed decomposition pathways [60]. The high number of edges under the *Ulva* diet resulted in many unique edges, 76% of total edges (Fig. 6h), but also in a significantly denser network and more sub-clusters of participating nodes than were seen in other diets (Additional file 1: Fig. S7d,e). Therefore, it is more likely that under the *Ulva* diet, any two microbes in the assembly will perform a direct association between themselves (most likely a positive one), as both the diameter and average path length were similar to those measured in other networks under different diets [61].

Our finding of a contradictory high-tail degree distribution under *Ulva* diet compared to other diets supports the advantage of the multi-parametric analysis for hub node identification as performed here. This analysis enabled the identification of an unassigned node as a hub microbe in the *Ulva* network as per its significant “high betweenness centrality” despite the somewhat lower “number of degrees”. The hub in the different networks included OTUs from six phyla of *Bacteroidetes* (3 OTUs of order *Bacteroidales*)*, Firmicutes* (2 OTUs of order *Clostridiales*), *Cyanobacteria* (2 OTUs of order *Gastranaerophilales*), *Proteobacteria* (1 OTU of genus *Photobacterium*)*, Spirochaetes* (1 OTU), *Tenericutes* (1 OTU of genus *Candidatus hepatoplasma*) and two more unassigned OTUs (Additional file 1: Table S6). Our identification of the hub nodes in the different networks as per the criteria for hub microbes [7] suggests Phylum *Bacteroidetes* as the keystone taxa, with a representative hub OTU in nearly all examined networks (Additional file 1: Table S6)*.* Our additional findings of the relatively high strain variability of *Bacteroidetes* (richness; a total of 111 OTUs, 2^nd^ highest) in the gut of *T. gratilla elatensis* sea urchin suggest further support for the importance of this keystone phylum. This strain variability revealed 3 core microbes, 7 specialist microbes (assigned to 5 different orders), and 4 *Ulva-*unique microbes (all assigned to genus *Roseimarinus),* all of this phylum.

Interestingly, the identified hub nodes in each network of the algal diets formed co-occurrence sub-clusters. Such a sub-cluster was also identified in the pellet-fed group but it included a fair number of co-exclusions between the hub nodes (Additional file 1: Fig. S8). Considering these findings, the synchronization between hub nodes in networks of the algal diets in terms of their co-occurrence may suggest a major role of these microbes in syntrophy/cross-feeding via complementary metabolisms [62]. Such syntrophy is greatly influenced by available resources (including feed) [63] and allows maintenance of functional redundancy in the community due to the presence of multiple taxa with shared metabolic function even when a particular node is eliminated from the network, [60, 64]. Under the algal diets tested here, this microbial syntrophy may be essential for the metabolism of the complex fibers that are the primary ingredients in these feeds [65, 66]. This could be compared, perhaps, with the syntrophic microbial metabolism of cellulases in cows’ rumens [67]. This is further supported by the fact that microbe connectivity in different regions of the gut was mainly through in-line/chain-form connections despite being spatially close in each of the examined regions.

Transfer of the sea urchins from the initial baseline algal diet of combined *U. fasciata* and *G. conferta* to the pellet diet treatment may have caused a more severe disturbance of their GMA than did the other two diet treatments in which the change was simply restriction to only one of these two algal species. This disturbance may have resulted in reduced diversity and a less dense network, consisting of low numbers of edges (also of exclusive ones), a fair number of co-exclusion associations, and fewer sub-clusters (Fig. 4b-d,h, Additional file 1: Fig. S7b,d,e). This perhaps indicates reduced stability and functionality of the bacterial community [6, 68] which may have resulted from switching the urchins to this new artificial diet made of ground-up land crops rather than natural marine feeds. A similar reduction was also measured in rodents when they were transferred to and from their natural feed as a result of periodic captivity [69], and in captive fish after rearing on an artificial pelleted feed [70].

**Fig. 5:**
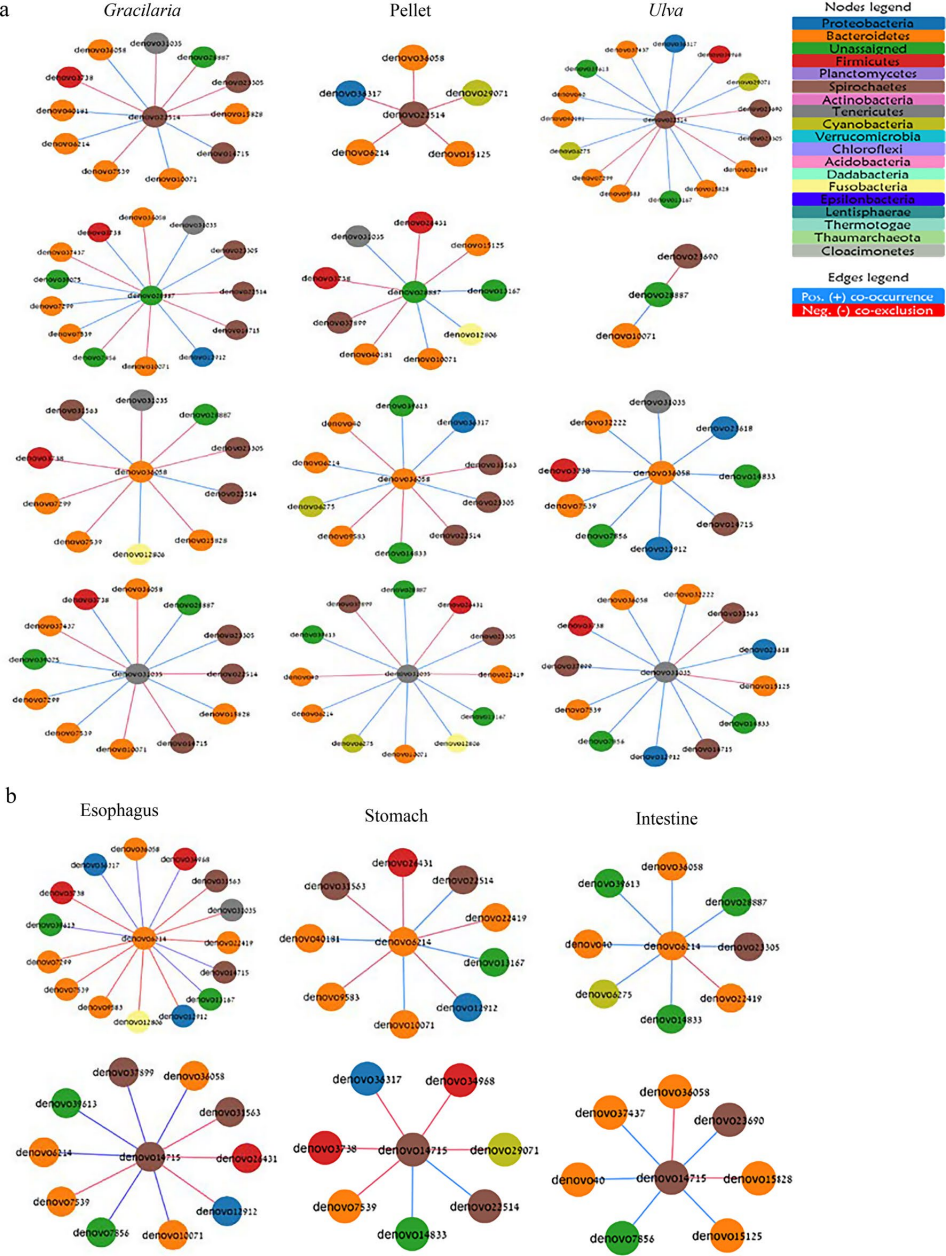
Driver nodes to rewire *T. gratilla elatensis* GMA association network under different variable**s** of diet and gut region. Driver microbes were extracted from networks using NetShift following significant change in degrees and associated partners. Only nodes that presented significant change as compared to the other two diets or regions were identified as driver microbes. Associations of driver nodes in **a** different diets or **b** gut regions. Annotation (genus level) of driver microbes include (upper to lower order): denovo22514 (*Spirochaeta2*), and denovo28887 (Unassigned) as driver microbes in the *Gracilaria*-diet network; denovo36058 (*Roseimarinus*) in the pelleted-diet network; denovo31035 (*Candidatus Hepatoplasma*) in the *Ulva*-diet network; denovo6214 (*Roseimarinus*) in the esophagus network; and denovo14715 (uncultured bacterium of *Spirochaetes*) in the intestine network. Nodes are colored following annotation at Phyla level (n = 54)

Network rewiring was greatest due to dietary influence, particularly between the pelleted and either of the algal diets, and less significant regarding the gut regions (Additional file 1: Fig. S9a–f). This discrepancy was also supported by the relatively high number of driver microbes that rewired diet-type networks as compared to their numbers in gut-region networks (Additional file 1: Table S7). Six microbes were identified as clear driver nodes due to their altered associations and higher centrality in only one particular niche (Fig. 5). The networks of the pelleted diet and esophagus were rewired by differing driver nodes of *Roseimarinus*, while driver nodes of *Sediminispirochaeta* or *C. hepatoplasma* rewired the intestine or *Ulva* network, respectively. The lack of associations of *Ulva-* driver node *C. hepatoplasma* with hub nodes in this network was significant as compared to other drivers found in other networks. Therefore, despite the highest occurrence and abundance of this single OTU of phylum *Tenericutes* in all niches and examined sea urchin individuals, we assume that *C. hepatoplasma* acts as a host-dependent node in the assembly, as was also suggested for this bacterial species in the case of another sea urchin [46]. Under the *Gracilaria* diet, network rewiring was driven by two nodes, one of *Spirochaetes* and another of an unassigned microbe. These two drivers revealed a competitive association with each other under this diet. Moreover, while the *Spichochaetes* driver revealed negative interaction with all hub nodes of *Gracilaria* network, the other driver node (unassigned) co-occurred with them. As compared to other drivers, the unassigned driver in *Gracilaria* network also revealed greatest exclusive edges, i.e., interaction with an exclusive partner or with the same partner but via opposite association. Although both hub and driver nodes reveal great centrality in the network, the high number of degrees characterized the former but not necessarily the latter. However, in some cases where the node creates exclusive degrees that also increase its overall connectivity, the microbe is identified as both hub and driver node in this niche. This was the case with the unassigned and *Roseimarinus* nodes in *Gracilaria* and esophagus networks, respectively (Fig. 5, Additional file 1: Table S6, S7).

### Generalism and specialism with regard to the potential metabolic functions of key microbes in sea urchin gut

**Fig. 6:**
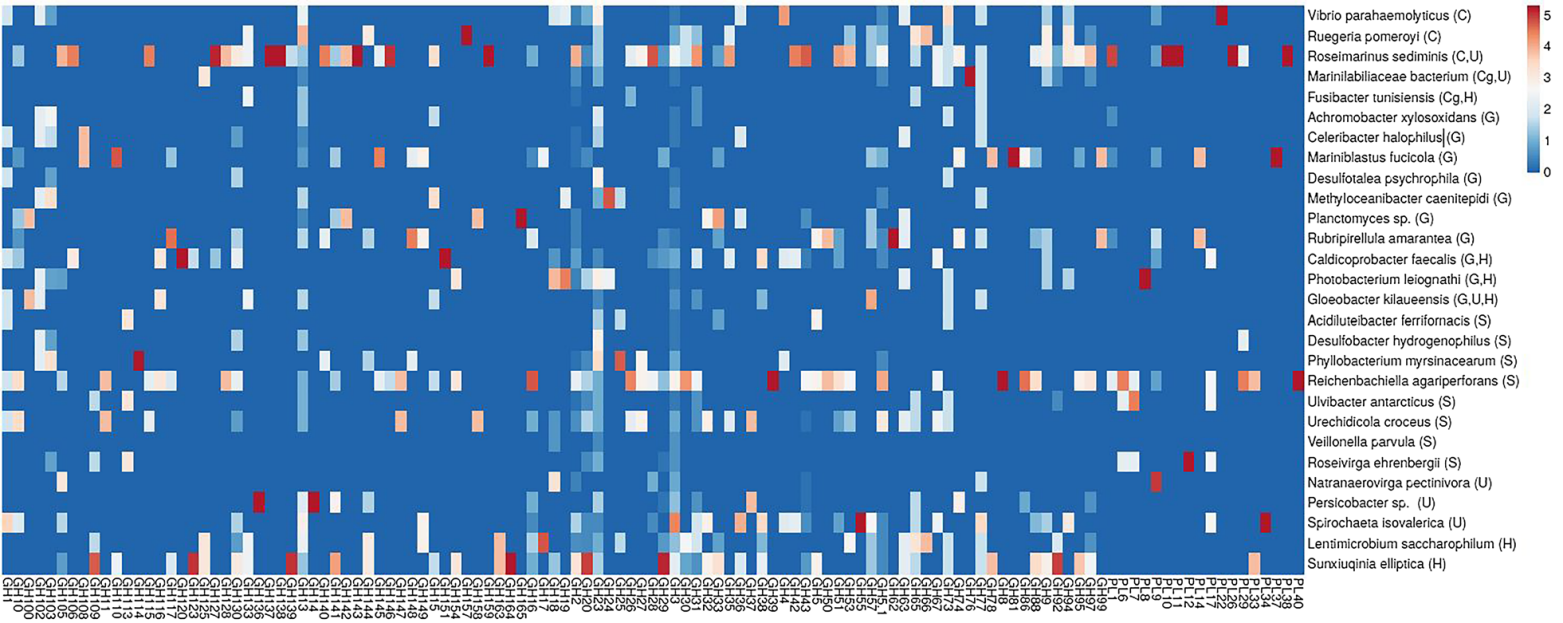
A heatmap diagram of the variation in content and number of copies of glycoside hydrolase (EC 3.2.1.-) and polysaccharide lyase (EC 4.2.2.-) genes in the genome of the identified key microbes that fell into the determination of core (C), core-generalist (Cg), generalist (G), specialist (S), or unique (U) microbes, and the keystone hub microbes in the microbial networks (H)

A primary concern in the current examination of the niche breadth, specialization, and networking of the microbes in T*. gratilla elatensis* gut was the validation of these results in terms of metabolic diversity. This has been highlighted as a key milestone that will improve our current understanding of the customary ecological definitions of generalism and specialism [51, 71, 72]. To do this, analysis was made of the potential metabolic repertoire in genomes of the key microbes identified in the gut, including the key hub, core, core-generalist, generalists, specialists, and the unique microbes, particularly concerning their richness in CAZymes. Some specific differences between the selected microbes were observed (Fig. 6, Additional file 1: Table S8) which can be attributed to a specific gut region or diet type, and in some cases also to a specific stage in the metabolism of dietary polysaccharides. Altogether, the genomes of the selected microbes presented a total of 271 carbohydrate-related enzyme classes (including sub-families) in the CAZy database. 167 of them were classified as glycoside hydrolases (GHs), 27 as polysaccharide lyases (PLs), 36 as glycoside transferase (GTs), 23 as carbohydrate binding modules, 12 as carbohydrate esterases (CEs), and 6 related to auxiliary activities. Of these classes, only GHs and PLs are involved in polysaccharide decomposition [73] and were further analyzed. Three bacterial strains of *Roseimarinus sediminis*, *Reichenbachiella agariperforans,* and *Sunxiuqinia elliptica* (all members of the phylum *Bacteroidetes* and isolated from marine environments) were richest in terms of the number of different CAZyme classes. These species were also richest in their content of CAZymes for polysaccharide decomposition, with 81 (*R. sediminis*) 73 (*R. agariperforans*), and 50 (*S. elliptica*) GHs and PLs. Examination of the genome of *R. sediminis* revealed 4 exclusive PL classes (10,11, 29, and 38), while among these classes both the PL29 and PL38 (characterized as chondroitin-sulfate-ABC endolyase and glucuronan lyase, respectively) presented the highest number of copies in the genome. These findings appear adequate for the correlation of the four OTUs which were unique to *Ulva-*fed sea urchins in the current research (i.e., *Ulva*-unique) and annotated as *R. sediminis*, to potential activity in the metabolism of *Ulva*-polysaccharides *in T. gratilla elatensis* gut. The rich content of CAZymes in the genome of *S. elliptica* agrees with the known capability of this fermentative microbe to decompose various carbohydrates such as D-fructose, L-rhamnose, D-xylose, myo-inositol, sorbitol, trehalose, D-mannitol, ribose, raffinose, gluconate, malonate, and propionate, each as a sole carbon source [74]. Hence, this characteristic may be attributed to our current finding of *R. sediminis* as a key hub node in the stomach of *T. gratilla elatensis*, where rapid fermentation is required for the metabolism of refractile and sulphated carbohydrates [48, 75, 76] such as those derived from decomposition of the complex polysaccharides in *Ulva* and *Gracilaria*. We also suggest additional members of *Bacteroidetes* identified here as correlated with the initial depolymerization stage in the metabolism of *Ulva* or *Gracilaria* to be key taxa in the gut of sea urchins fed these diets. Among these is *R. agariperforans* which consists both of ulvan lyases (PL40 and PL37) and β-agarases (GH50 and GH86) that are correlated with degradation of *Ulva*-ulvan [77] and *Gracilaria*-agarose [78], respectively. The β-agarase GH86 is also found in another key species, *Persicobacter*. (referred to here as denovo796; also from the *Bacteroidetes*) [79], which was correlated in the current study with the *Gracilaria*-fed sea urchins (*Gracilaria*-unique). However, it should be noted that low CAZyme content in a genome is not necessarily singular evidence for the non-engagement of a microbe in polysaccharide metabolism. A potential example gleaned from this study may be the core-generalist *Fusibacter tunisiensis* (referred to here as denovo 3738) which consists only of 7 CAZymes (with a total of 11 copies) but presents a relatively unique characteristic in terms of energy gain from the reduction of thiosulfate and elemental sulfur into sulfide. This metabolism is part of the sulfur cycle in anaerobic salty environments such as marine sediment [80], while in the currently examined sea urchins it may be attributed to the metabolism of the algal polysaccharides. This was evident in previous studies on decomposition of *Ulva* and *Gracilaria* where sulfate-reducing bacteria were noted for their major role in the process [81, 82].

While the above results revealed some supportive evidence for the specialization of the diet-unique microbes in terms of their unique metabolic content as well, it is still questionable whether encompassing a broad or narrow niche in the gut of *T. gratilla elatensis* is indicative of microbes’ own metabolic arsenal (and vice versa). An interesting finding concerning this is the core microbe *Candidatus hepatoplasma* (a single OTU annotated as *C. Hepatoplasma crinochetorum Av*) identified here. This microbe, in spite of being fairly abundant in all examined niches of different diets and gut regions, reveals a relatively small genome of only 657,101 bp [83], no CAZymes, and also lacks a cell wall. This reinforces our previous assumption of this bacterium’s host-dependent lifestyle in the gut of sea urchins. Another common microbe in *T. gratilla elatensis* gut was the generalist *Gloeobacter kilaueensis* (*Cyanobacteria*) which was found in all the niches examined. Such generalism in terms of a wide niche breadth can be correlated in the current study with the photoautotrophic lifestyle of this microbe [84] essential to its success in the sea urchin’ light-penetrated gut, independent of the variation in available nutrients in the ingesta from different regions or diet types. The latter may be supported also by the relatively low content of CAZymes in this microbe’s genome. In contrast to these two microbes, nearly all the other microbes that occupied broad niches, i.e., core, generalists, and core-generalists, revealed a relatively rich carbohydrate metabolic capacity of between 14 and 81 CAZymes. Specialist microbes, with only two exceptions (*R. agariperforans* and *U. croceus*), presented a relatively poorer carbohydrate metabolism potential consisting of between 7 and 13 enzyme classes (including sub-families). This finding may emphasize the correlation between a narrow niche breadth in terms of occupancy and occurrence, as measured for these microbes in the current study, and specification in the niche in terms of carrying only relevant enzymes for carbohydrates metabolism which are vital for gaining energy from the here examined diets.

## Conclusions

The mono-specific algal diet altered the gut microbial assembly of *T. gratilla elatensis *in terms of composition, networking, and potential metabolism. A dense and well-connected microbial network was measured in the *Ulva-*fed sea urchins and may suggest, together with the animal's rapid growth, that this alga was most nutritious among the experimental diets. Generalism and specialism of the microbes in the gut agreed with their richness in CAZymes, while specialization in narrower niches of diet types was evident in terms of both occurrence and the specific metabolic arsenal the microbe carries that may aid the host in nutrition. Finally, we conclude *Bacteroidetes* to be the keystone phylum in the gut of *T. gratilla elatensis* with many different strains inhabiting this organ. Among are key hub microbes in the niche-specific networks, core microbes that occupied nearly all niches, generalist microbes that were suited to broader niches, as they are rich in their CAZyme content for decomposition of various polysaccharides, and specialist microbes that were correlated with the initial depolymerization stage in the metabolism of *Ulva* or *Gracilaria*.

## Supplementary Information

**Additional file 1**.** Figure S1**. The dissection process of sea urchin* T. gratilla elatensis* for sampling of the three major regions of the digestive tract. Individual sea urchin (a); circumference cutting presenting upper and lower coelom (b); coelom after removal of the digestive tract (c); (d) Unfolded digestive tract presenting (left to right) the esophagus, stomach, and intestine regions. Red arrow indicates the Aristotle's lantern.** Figure S2**. Rarefaction curves present the observed OTUs in* T. gratilla elatensis* GMA from each dietary treatment and gut region (n = 51).** Figure S3**. Non-metric multidimensional scaling (NMDS) based on Bray-Curtis dissimilarities displaying GMA in different gut regions of* T. gratilla elatensis* fed with* Gracilaria* (a), algal-free pellets (b), or* Ulva* (c). Each dot represents GMA in a specific gut region in an individual sea urchin and is colored in red, green, and blue for esophagus, intestine, and stomach, respectively. The overall area of GMA in each gut region was measured following differences between individuals (inter-niche differences) and indicates overlaps between gut regions (n = 51).** Figure S4**. (a). A bubble chart of the 17 taxonomic phyla that were identified in the sea urchin digestive tract. The cumulative abundance of all OTUs of each phylum are represented as circles, each representing the sized cumulative abundance of the phylum OTUs in one individual sea urchin under a given treatment of diet and gut region. (b). Abundance of selected phyla with significant variations in the different examined niches of gut region and diet. Differences between individual samples in each specific niche are shown as box plots where middle line in the box indicates mean value, error lines present SD, and whiskers are drawn from the 10th to 90th percentiles (n = 54).** Figure S5**. Abundance/occurrence ratio of microbes identified as core, core generalists, generalists, specialists, or unique. Ratios of all individuals in group are summarized and presented as box plots with middle line for median, plus sign for mean, error lines for SD, and whiskers drawn from the 10th to 90th percentiles (n = 50).** Figure S6**. Degrees distribution in GMA networks reveals the different distribution patterns (Poisson or Law-tail) of degrees between nodes in the microbe associations in the following niches: general network (a), under different diets of* Gracilaria* (b), pellets (c) or* Ulva* (d); or in different gut regions of esophagus (e), stomach (f), or intestine (g).** Figure S7**. Topology indices of the GMA association networks examined under different variables of diet (left) or gut region (right). Indices of the general network are shown in graph sets of different diets (left graphs).** Figure S8**. Schematic diagram of the associations of hub nodes in networks of different diets and gut regions. Type of association (co-occurrence or co-exclusion in blue or red lines, respectively) is also shown in cases of hub-hub association. Nodes are colored as per annotation at phyla level.** Figure S9**. Pairwise analysis of the association networks in different niches. Each network represents a network pair which was analyzed as control versus case in the respective order for network pairs:* Gracilaria* vs. pellet (a);* Gracilaria* vs.* Ulva* (b);* Ulva* vs. pellet (c); esophagus vs. stomach (d); esophagus vs. intestine (e); and stomach vs. esophagus (f); Edges in the combined network are colored as per their affiliation as exclusive to the control network (green), exclusive to case network (red), or shared between both (blue). Nodes in networks present only the identified driver microbes as measured by NetShift following their significant increased betweenness in the ‘case’ (red nodes) or control network (black nodes). Nodes diameter indicates NESH score from smaller to higher.** Table S1**. Ingredients in the formulated algal-free, plant-based pellets for* T. gratilla elatensis* used in current study.** Table S2**. Physical characteristics of wet weight and diameter (mean ± SE) of sea urchin individuals after culture under the different diet- types of* Ulva*, or* Gracilaria*, or pellets. Values are mean ± SE (n=3). n.d. = not determined.** Table S3**. OTU count and number of reads for each of the examined samples from a particular gut region and diet regime (n = 54).** Table S4**. OTU count for the various identified phyla in sea urchin gut samples (n = 54).** Table S5**. Shared edges between GMA association networks in different diets or gut regions (n = 54) indicating type of relationship (co-occurrence or co-exclusion) and the participating nodes.** Table S6**. Topology properties of hub microbes in GMA association networks presenting number of degrees, closeness, and betweenness indices in the examined network of the different diets or gut regions (n = 54).** Table S7**. Topology properties of identified driver nodes in GMA association networks presenting Jaccard Edge Index, NESH score and delta betweenness centrality (normalized to the average path length) indices as measured in pairwise analyses of control versus case networks. Real drivers, i.e. nodes that were identified as driver in a specific niche against each of two other niches, are indicated in bold.** Table S8**. BLAST (Basic Local Alignment Search Tool) results of the closest phylogenetic species that was identified for each of the OTUs that were identified in current research as core, core-generalist, generalist, specialist, unique, or hub microbes. The bio project number in the NCBI database, where the genome of any of the assigned species is available, is provided. Not found = no genome was available for the closest phylogenetically-related species.

## Data Availability

The generated datasets in this study are accessible through ENA with the accession number PRJEB42313. Supplementary data and material are provided.

## Abbreviations

CAZy/CAZymes: Carbohydrate active enzymes
FDR: False discovery rate
GH: Glycoside hydrolase
GMA: Gut-microbe assembly
JEI: Jaccard edge index
NCM: National Center for Mariculture
PL: Polysaccharide lyase
QIIME: Quantitative insights into microbial ecology
NESH: Neighbor shift score
LEfSe: Linear discriminant analysis effect size
CCLasso: Correlation interference for compositional data through lasso
OTU: Operational taxonomic unit
PERMANOVA: Permutational multivariate analysis of variance
