# Supplementary material for: Mono-specific algal diets shape microbial networking in the gut of the sea urchin Tripneustes gratilla elatensis
Source: Anim Microbiome. 2021 Nov 15;3:79. doi: 10.1186/s42523-021-00140-1 (PMC8594234; doi:10.1186/s42523-021-00140-1)
Supplement: Supplementary file 1 — Additional file 1. Figure S1. The dissection process of sea urchin T. gratilla elatensis for sampling of the three major regions of the digestive tract. Individual sea urchin (a); circumference cutting presenting upper and lower coelom (b); coelom after removal of the digestive tract (c); (d) Unfolded digestive tract presenting (left to right) the esophagus, stomach, and intestine regions. Red arrow indicates the Aristotle's lantern. Figure S2. Rarefaction curves present the observed OTUs in T. gratilla elatensis GMA from each dietary treatment and gut region (n = 51). Figure S3. Non-metric multidimensional scaling (NMDS) based on Bray-Curtis dissimilarities displaying GMA in different gut regions of T. gratilla elatensis fed with Gracilaria (a), algal-free pellets (b), or Ulva (c). Each dot represents GMA in a specific gut region in an individual sea urchin and is colored in red, green, and blue for esophagus, intestine, and stomach, respectively. The overall area of GMA in each gut region was measured following differences between individuals (inter-niche differences) and indicates overlaps between gut regions (n = 51). Figure S4. (a). A bubble chart of the 17 taxonomic phyla that were identified in the sea urchin digestive tract. The cumulative abundance of all OTUs of each phylum are represented as circles, each representing the sized cumulative abundance of the phylum OTUs in one individual sea urchin under a given treatment of diet and gut region. (b). Abundance of selected phyla with significant variations in the different examined niches of gut region and diet. Differences between individual samples in each specific niche are shown as box plots where middle line in the box indicates mean value, error lines present SD, and whiskers are drawn from the 10th to 90th percentiles (n = 54). Figure S5. Abundance/occurrence ratio of microbes identified as core, core generalists, generalists, specialists, or unique. Ratios of all individuals in group are summarized an [file 42523_2021_140_MOESM1_ESM.pdf]

Supplementary material

Figures

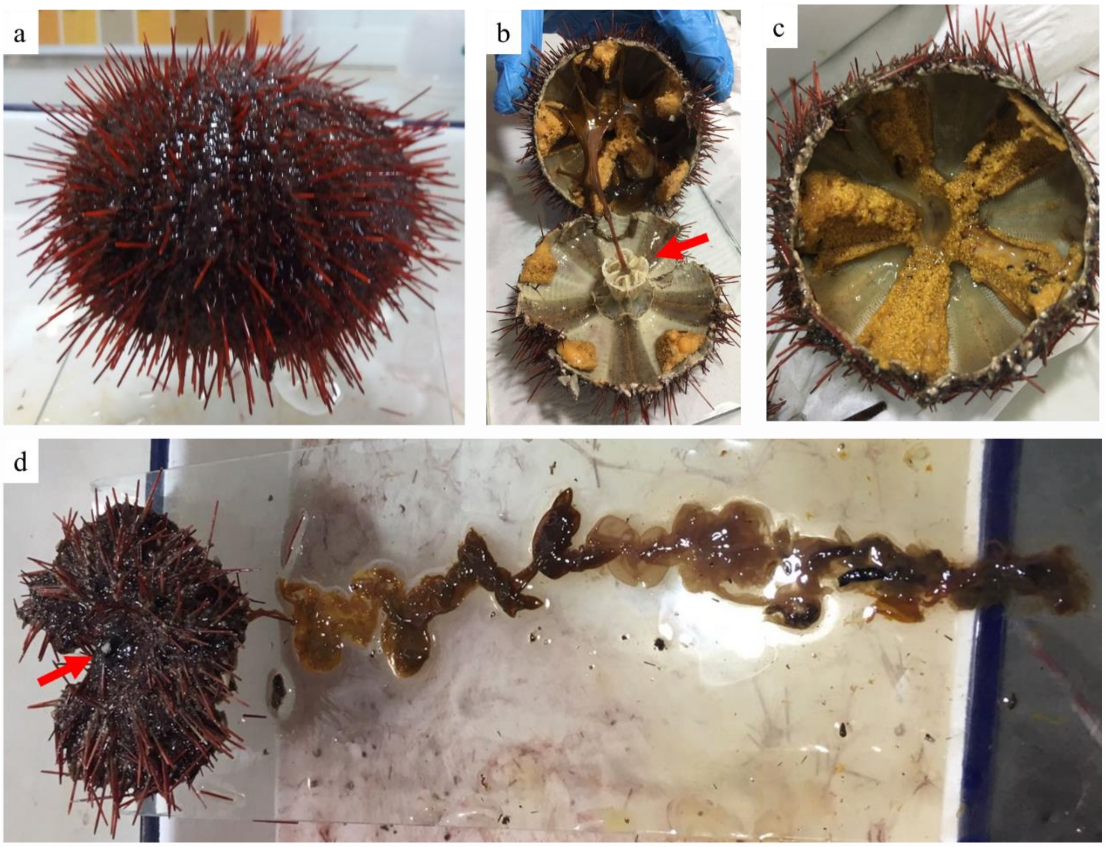

Figure S1. The dissection process of sea urchin *T. gratilla elatensis* for sampling of the three major regions of the digestive tract. Individual sea urchin (a); circumference cutting presenting upper and lower coelom (b); coelom after removal of the digestive tract (c); (d) Unfolded digestive tract presenting (left to right) the esophagus, stomach, and intestine regions. Red arrow indicates the Aristotle's lantern.

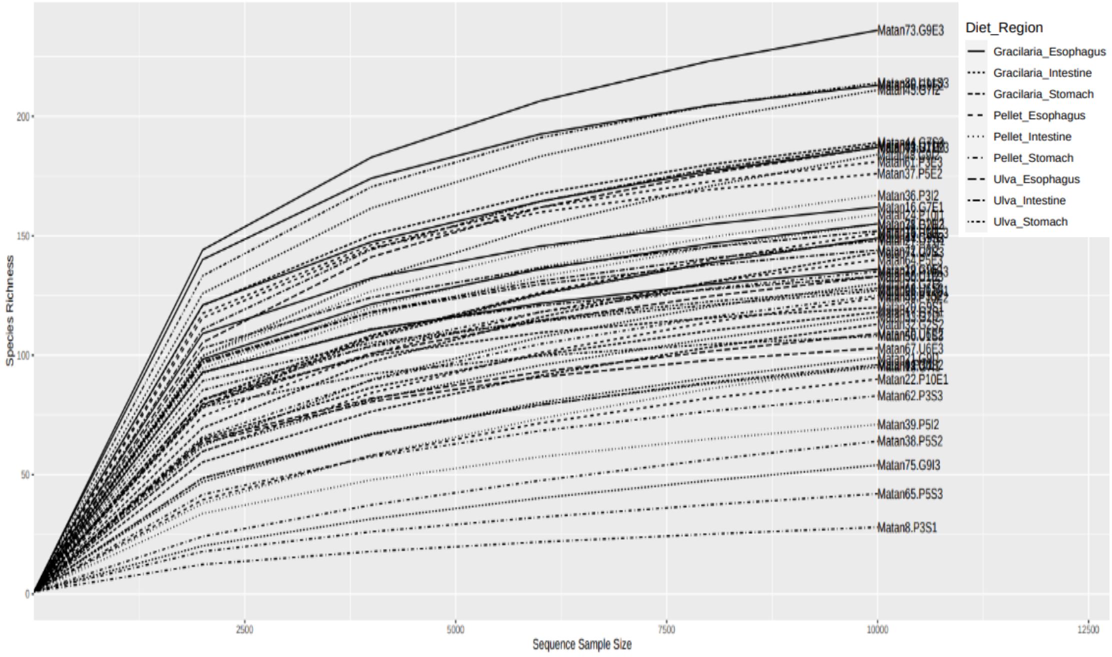

Figure S2. Rarefaction curves present the observed OTUs in *T. gratilla elatensis* GMA from each dietary treatment and gut region (n=51).

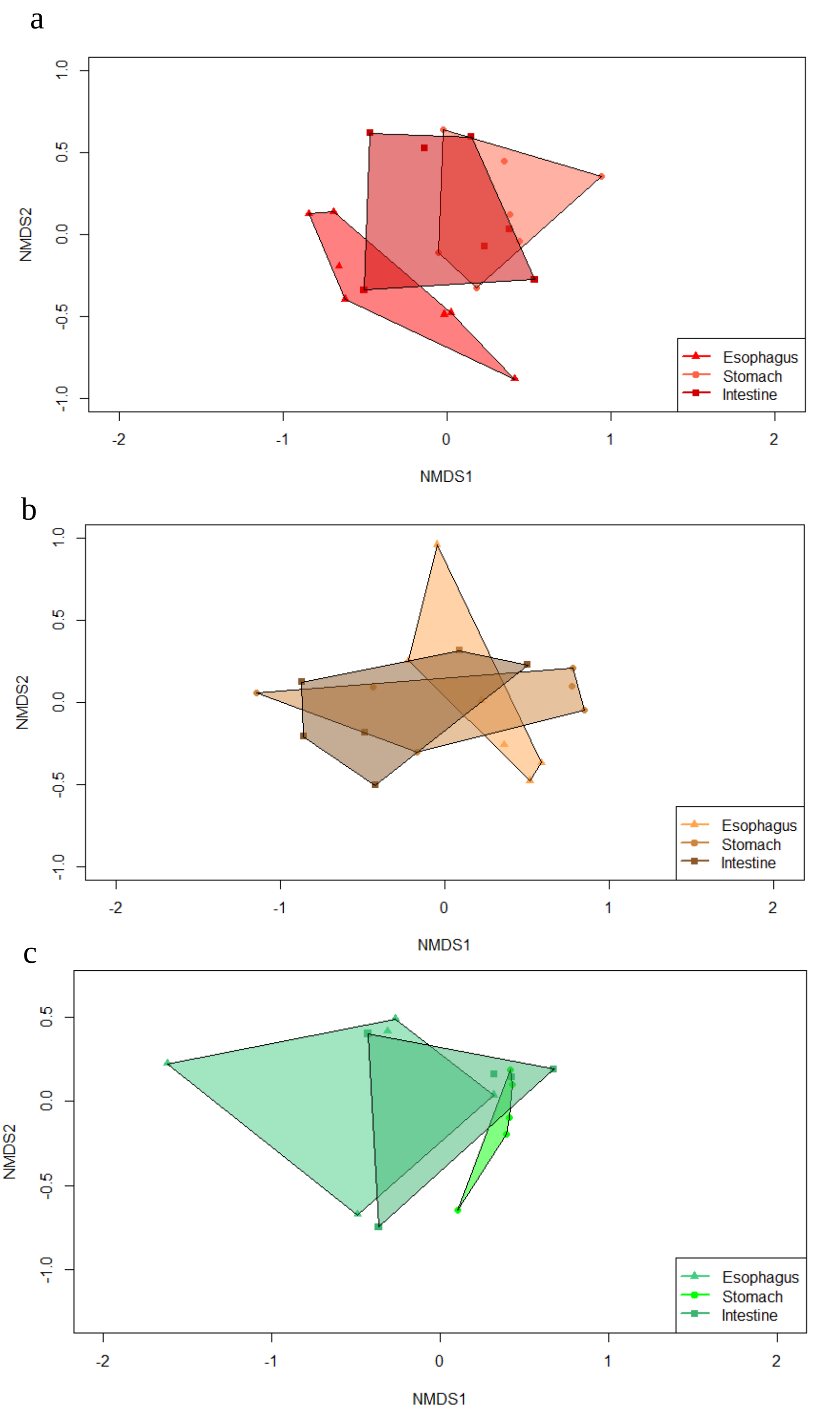

Figure S3. Non-metric multidimensional scaling (NMDS) based on Bray-Curtis dissimilarities displaying GMA in different gut regions of *T. gratilla elatensis* fed with *Gracilaria* (a), algal-free pellets (b), or *Ulva* (c). Each dot represents GMA in a specific gut region in an individual sea urchin and is colored in red, green, and blue for esophagus, intestine, and stomach, respectively. The overall area of GMA in each gut

region was measured following differences between individuals (inter-niche differences) and indicates overlaps between gut regions (n=51).

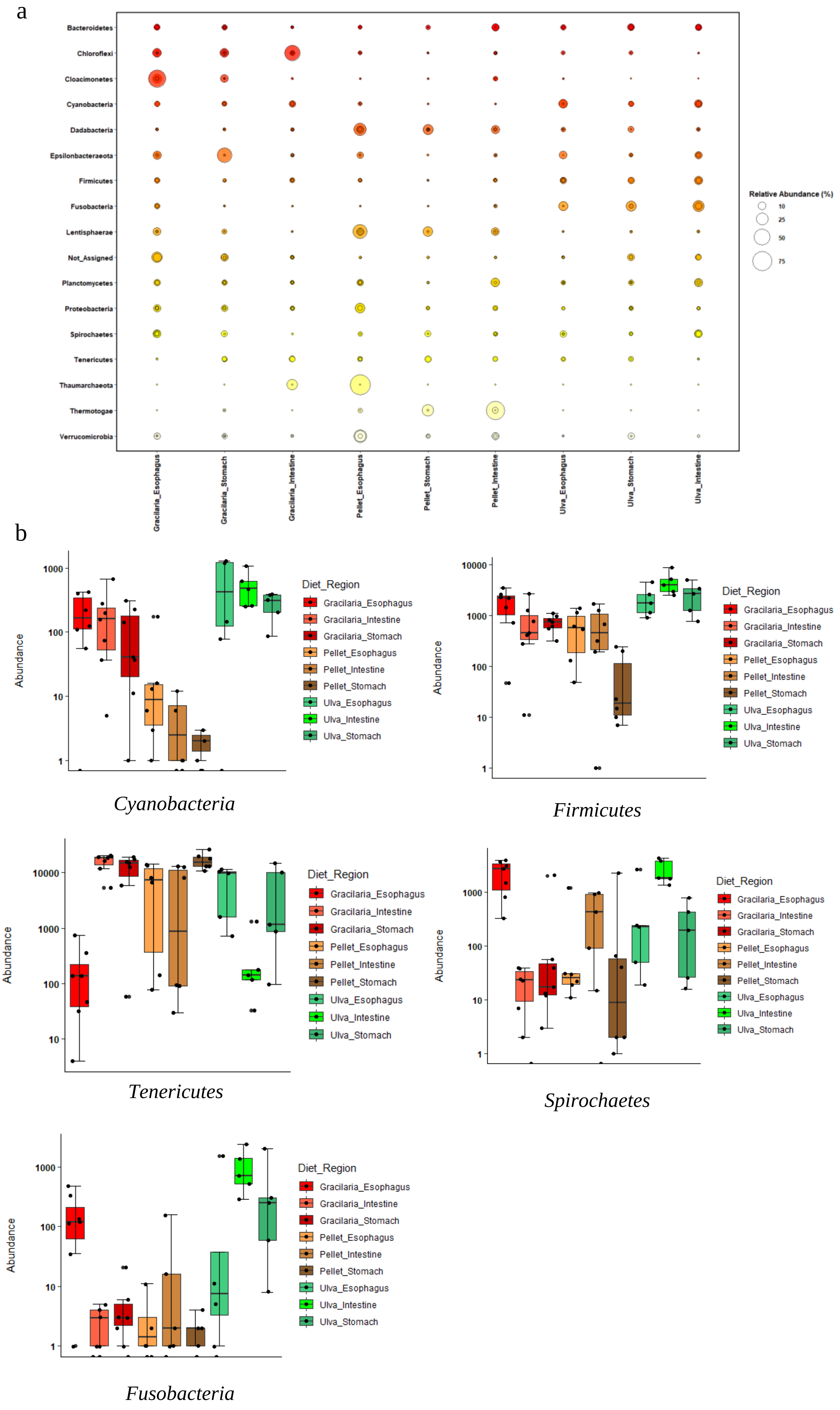
a
Bacteroidetes
Chloroflexi
Cloacimonetes
Cyanobacteria
Dadabacteria
Epsilonbacteraeota
Firmicutes
Fusobacteria
Lentisphaerae
Not_Assigned
Planctomycetes
Proteobacteria
Spirochaetes
Tenericutes
Thaumarchaeota
Thermotogae
Verrucomicrobia
Gracilaria_Esophagus
Gracilaria_Stomach
Gracilaria_Intestine
Pellet_Esophagus
Pellet_Stomach
Pellet_Intestine
Ulva_Esophagus
Ulva_Stomach
Ulva_Intestine
Relative Abundance (%)
10
25
50
75
b
Abundance
Diet_Region
Gracilaria_Esophagus
Gracilaria_Intestine
Gracilaria_Stomach
Pellet_Esophagus
Pellet_Intestine
Pellet_Stomach
Ulva_Esophagus
Ulva_Intestine
Ulva_Stomach
Cyanobacteria
Firmicutes
Tenericutes
Spirochaetes
Fusobacteria

Figure S4. (a). A bubble chart of the 17 taxonomic phyla that were identified in the sea urchin digestive tract. The cumulative abundance of all OTUs of each phylum are represented as circles, each representing the sized cumulative abundance of the phylum OTUs in one individual sea urchin under a given treatment of diet and gut region. (b). Abundance of selected phyla with significant variations in the different examined niches of gut region and diet. Differences between individual samples in each specific niche are shown as box plots where middle line in the box indicates mean value, error lines present SD, and whiskers are drawn from the 10th to 90th percentiles (n=54).

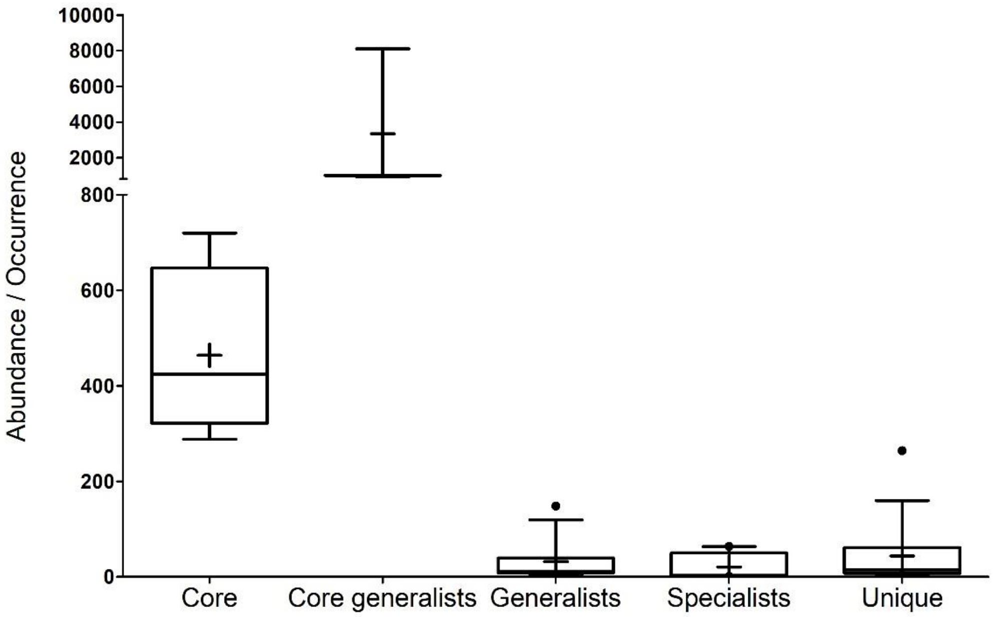

Figure S5. Abundance/occurrence ratio of microbes identified as core, core generalists, generalists, specialists, or unique. Ratios of all individuals in group are summarized and presented as box plots with middle line for median, plus sign for mean, error lines for SD, and whiskers drawn from the 10th to 90th percentiles (n=50).

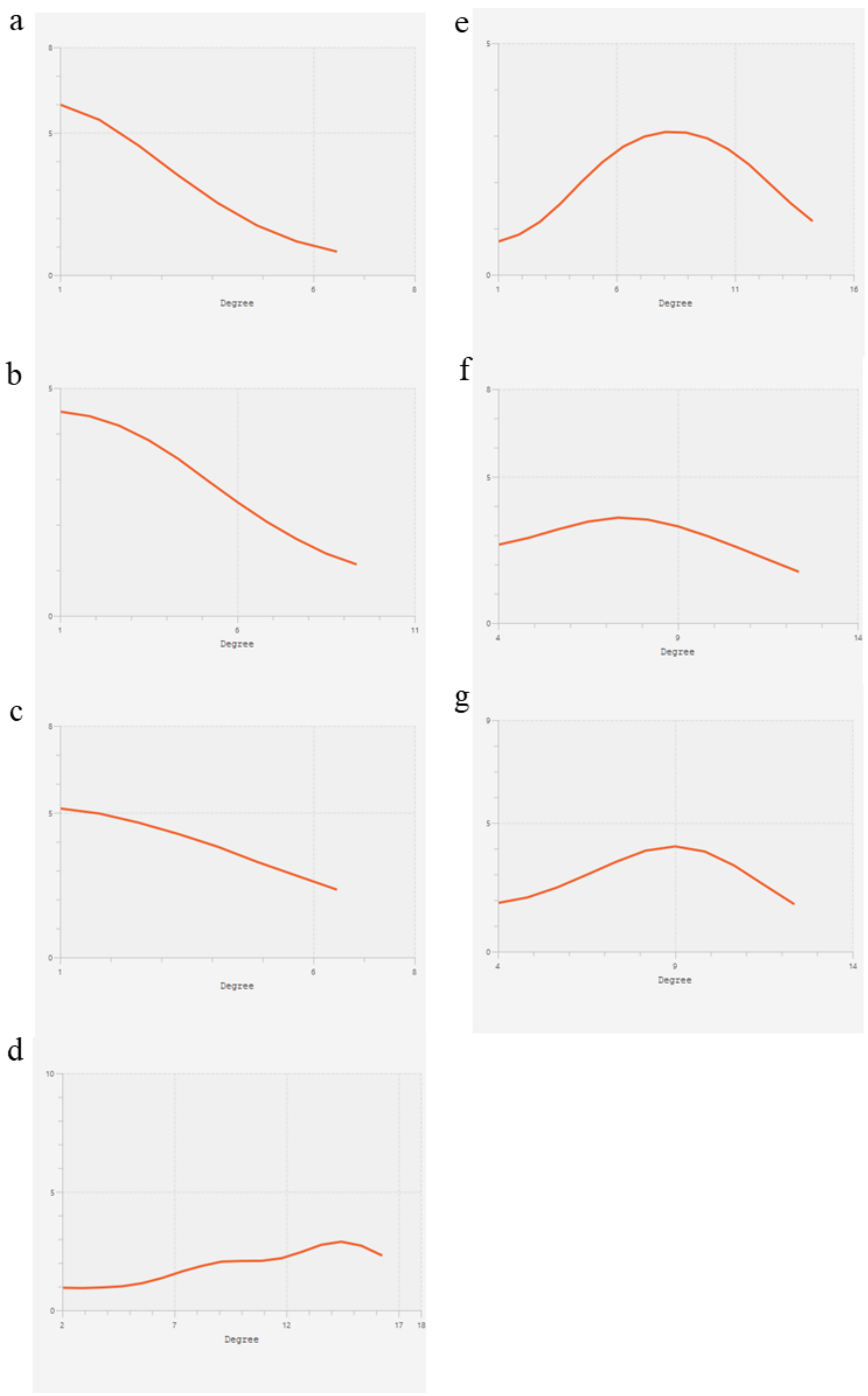

Figure S6. Degrees distribution in GMA networks reveals the different distribution patterns (Poisson or Law-tail) of degrees between nodes in the microbe associations in the following niches: general network (a), under different diets of *Gracilaria* (b), pellets (c) or *Ulva* (d); or in different gut regions of esophagus (e), stomach (f), or intestine (g).

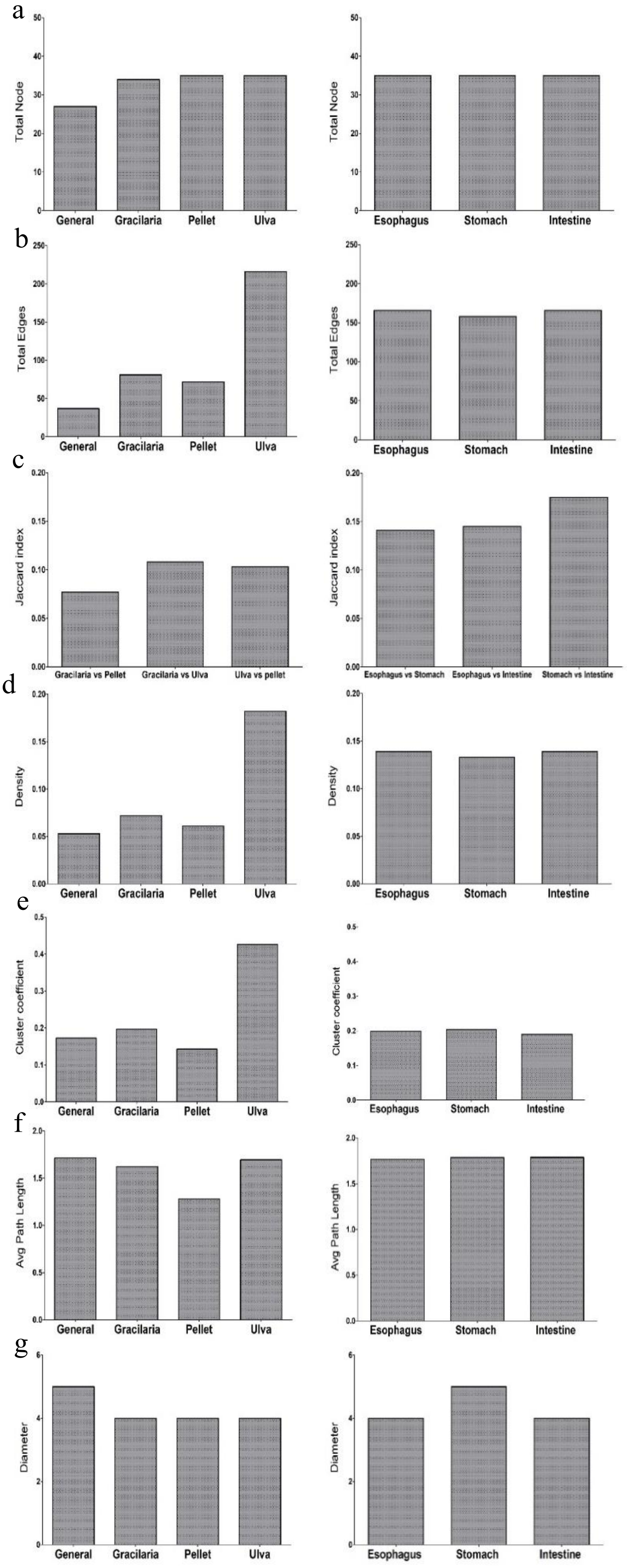
a
Total Node
General
Gracilaria
Pellet
Ulva
Esophagus
Stomach
Intestine
b
Total Edges
c
Jaccard index
Gracilaria vs Pellet
Gracilaria vs Ulva
Ulva vs pellet
Esophagus vs Stomach
Esophagus vs Intestine
Stomach vs Intestine
d
Density
e
Cluster coefficient
f
Avg Path Length
g
Diameter

Figure S7: Topology indices of the GMA association networks examined under different variables of diet (left) or gut region (right). Indices of the general network are shown in graph sets of different diets (left graphs).

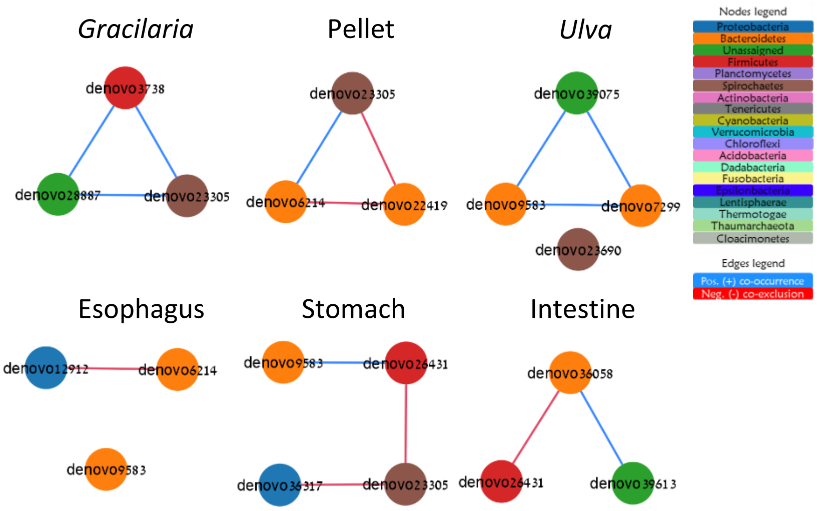

Figure S8: Schematic diagram of the associations of hub nodes in networks of different diets and gut regions. Type of association (co-occurrence or co-exclusion in blue or red lines, respectively) is also shown in cases of hub-hub association. Nodes are colored as per annotation at phyla level.

a

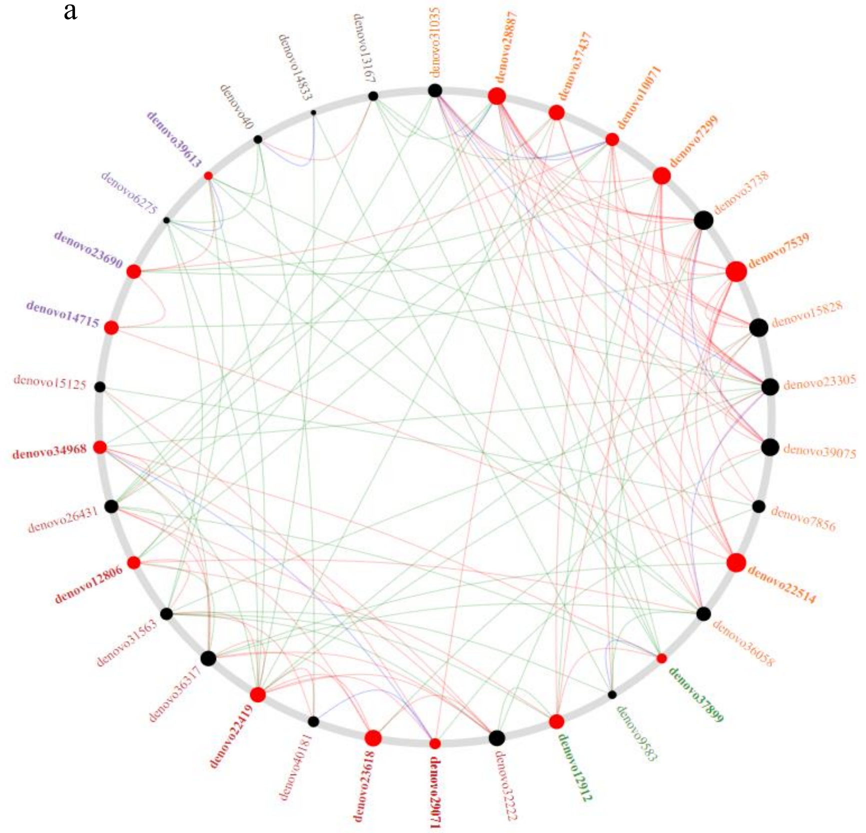
denovo31035
denovo28887
denovo37437
denovo10071
denovo7299
denovo3738
denovo7539
denovo15828
denovo23305
denovo39075
denovo7856
denovo22514
denovo36058
denovo37899
denovo9583
denovo12912
denovo32222
denovo29071
denovo23618
denovo40181
denovo22419
denovo36317
denovo31563
denovo12806
denovo26431
denovo34968
denovo15125
denovo14715
denovo23690
denovo6275
denovo39613
denovo40
denovo14833
denovo13167

b

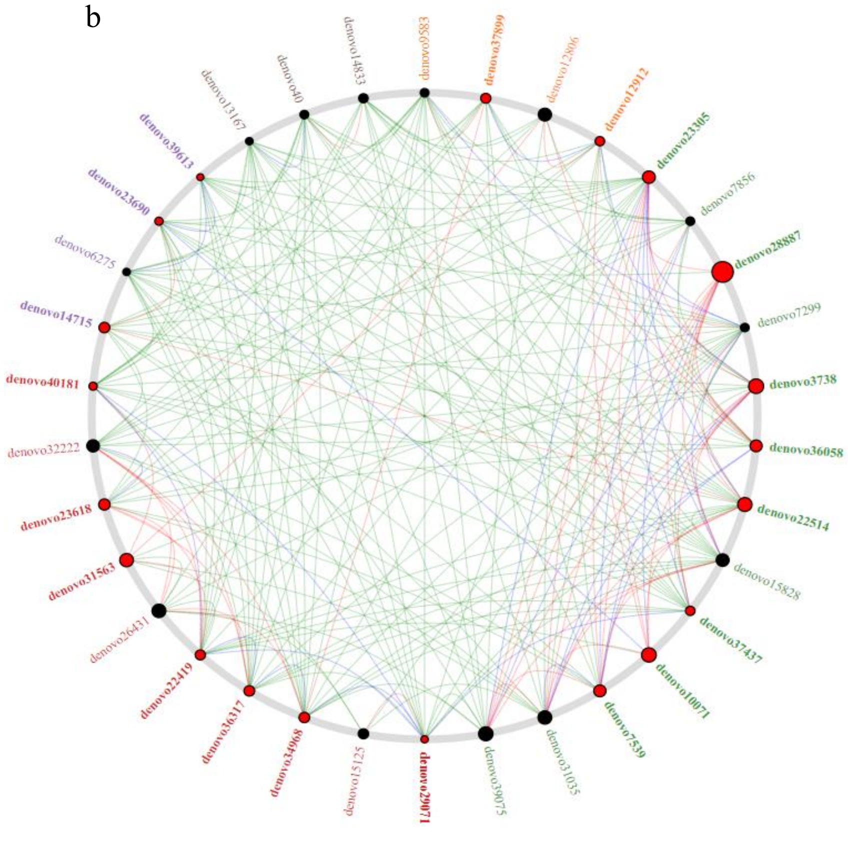
denovo9583
denovo37899
denovo12806
denovo12912
denovo23305
denovo7856
denovo28887
denovo7299
denovo3738
denovo36058
denovo22514
denovo15828
denovo37437
denovo10071
denovo7539
denovo31035
denovo39075
denovo29071
denovo15125
denovo34968
denovo36317
denovo22419
denovo26431
denovo31563
denovo23618
denovo32222
denovo40181
denovo14715
denovo6275
denovo23690
denovo39613
denovo13167
denovo40
denovo14833

c

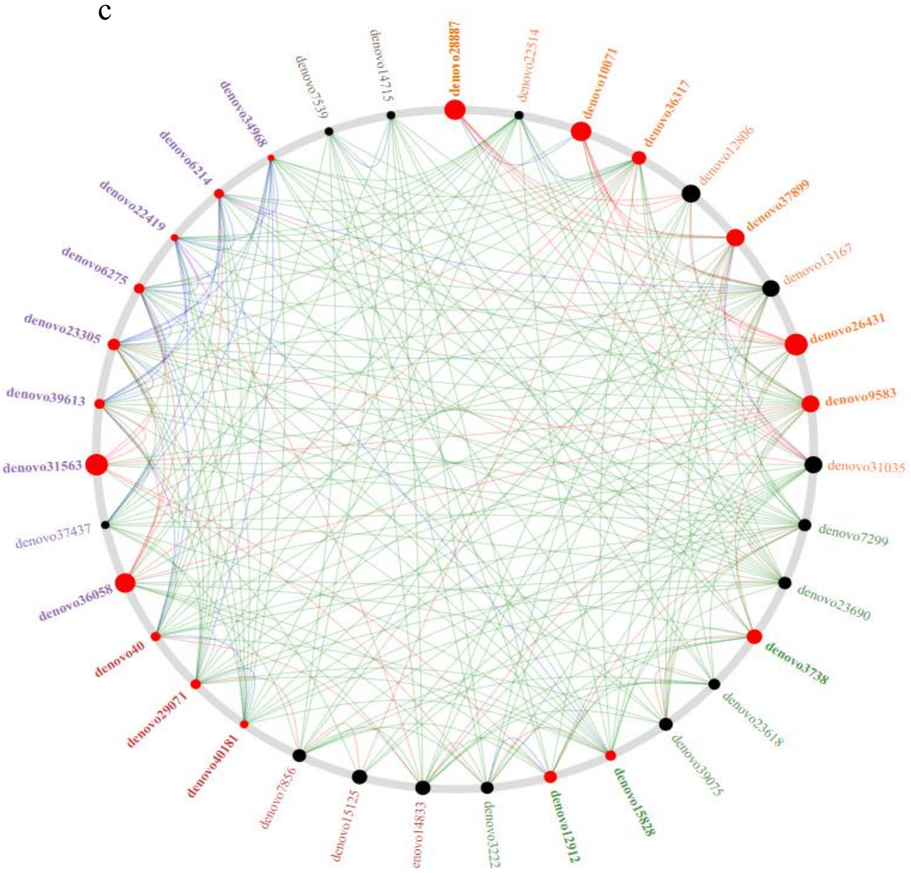
denovo28887
denovo22514
denovo10071
denovo36317
denovo12806
denovo37899
denovo13167
denovo26431
denovo9583
denovo31035
denovo7299
denovo23690
denovo3738
denovo23618
denovo39075
denovo15828
denovo12912
denovo3222
enovo14833
denovo15125
denovo7856
denovo40181
denovo29071
denovo40
denovo36058
denovo37437
denovo31563
denovo39613
denovo23305
denovo6275
denovo22419
denovo6214
denovo34968
denovo7539
denovo14715

d

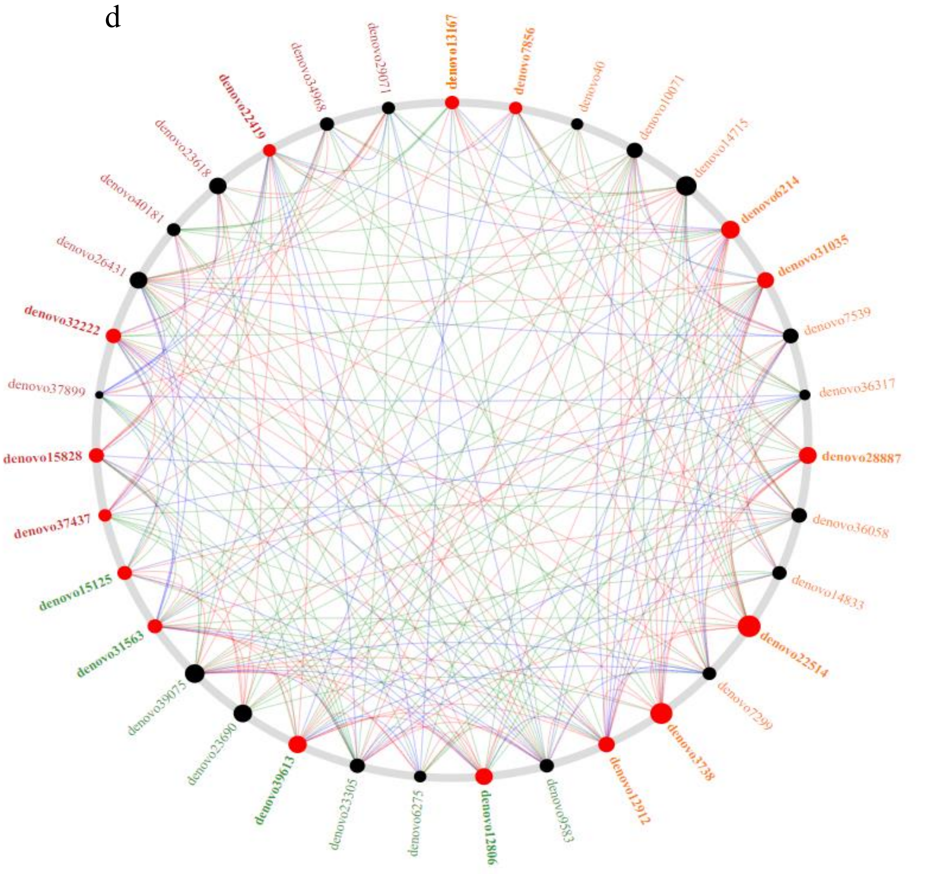
denovo13167
denovo7856
denovo40
denovo10071
denovo14715
denovo6214
denovo31035
denovo7539
denovo36317
denovo28887
denovo36058
denovo14833
denovo22514
denovo7299
denovo3738
denovo12912
denovo9583
denovo12806
denovo6275
denovo23305
denovo39613
denovo23690
denovo39075
denovo31563
denovo15125
denovo37437
denovo15828
denovo37899
denovo32222
denovo26431
denovo40181
denovo23618
denovo22419
denovo34968
denovo29071

e

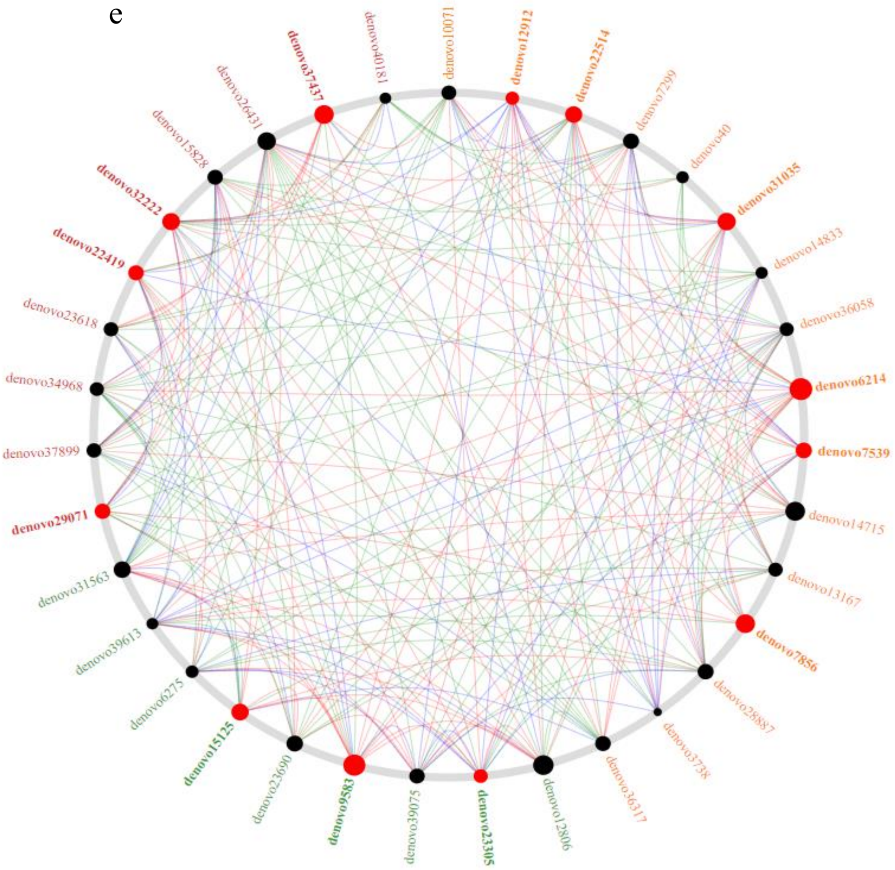
denovo40181
denovo10071
denovo12912
denovo22514
denovo7299
denovo40
denovo31035
denovo14833
denovo36058
denovo6214
denovo7539
denovo14715
denovo13167
denovo7856
denovo28887
denovo3738
denovo36317
denovo12806
denovo23305
denovo39075
denovo9583
denovo23690
denovo15125
denovo6275
denovo39613
denovo31563
denovo29071
denovo37899
denovo34968
denovo23618
denovo22419
denovo32222
denovo15828
denovo26431
denovo37437

f

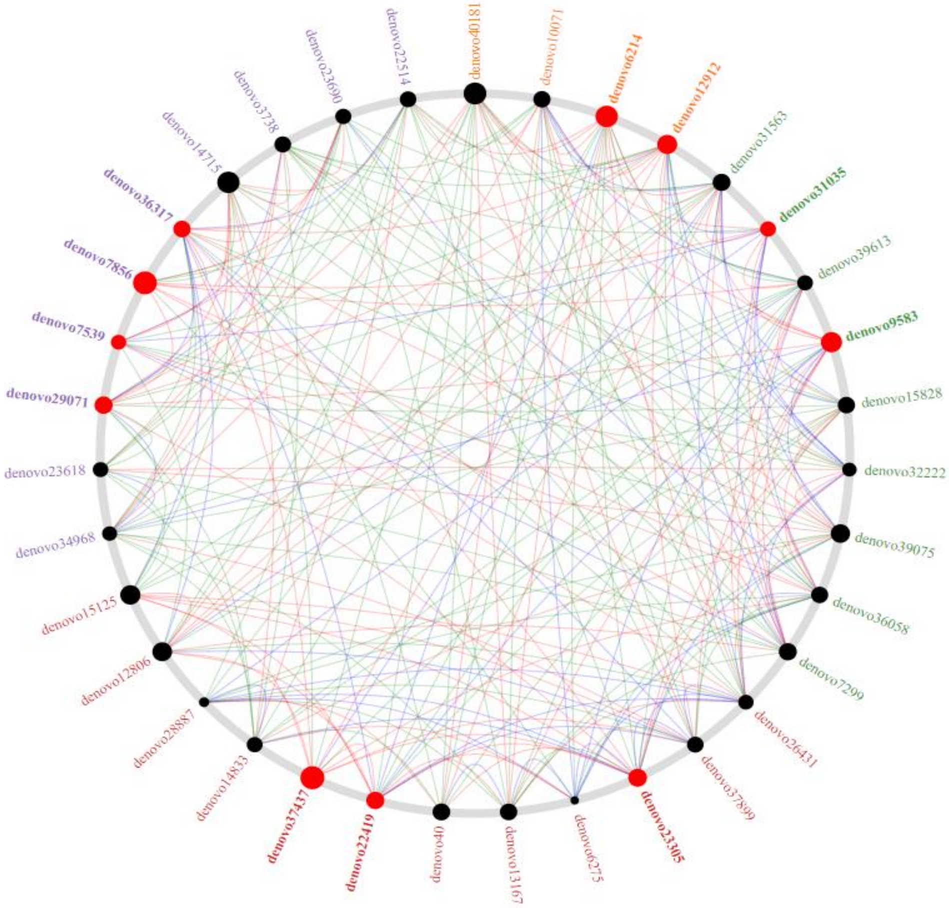
denovo40181
denovo10071
denovo6214
denovo12912
denovo31563
denovo31035
denovo39613
denovo9583
denovo15828
denovo32222
denovo39075
denovo36058
denovo7299
denovo26431
denovo37899
denovo23305
denovo6275
denovo13167
denovo40
denovo22419
denovo37437
denovo14833
denovo28887
denovo12806
denovo15125
denovo34968
denovo23618
denovo29071
denovo7539
denovo7856
denovo36317
denovo14715
denovo3738
denovo23690
denovo22514

Figure S9: Pairwise analysis of the association networks in different niches. Each network represents a network pair which was analyzed as control versus case in the respective order for network pairs: *Gracilaria* vs. pellet (a); *Gracilaria* vs. *Ulva* (b); *Ulva* vs. pellet (c); esophagus vs. stomach (d); esophagus vs. intestine (e); and stomach vs. esophagus (f); Edges in the combined network are colored as per their affiliation as exclusive to the control network (green), exclusive to case network (red), or shared between both (blue). Nodes in networks present only the identified driver microbes as measured by NetShift following their significant increased betweenness in the 'case' (red nodes) or control network (black nodes). Nodes diameter indicates NESH score from smaller to higher.

Tables

Table S1. Ingredients in the formulated algal-free, plant-based pellets for *T. gratilla elatensis* used in current study.

Ingredient	Dry weight (gr)
Whole-wheat	335.9
Rapeseed	210
Soya pulp	180
Corn starch	150
Calcium carbonate	20
Minerals	15
Choline chloride	4
Vitamins mixture	4
Vitamin C	0.6
Vitamin E	0.5
Lecithin	20
Linseed oil	20
Gelatin	40
Total	1000

Table S2. Physical characteristics of wet weight and diameter (mean ± SE) of sea urchin individuals after culture under the different diet- types of *Ulva*, or *Gracilaria*, or pellets. Values are mean ± SE (n=3). n.d. = not determined.

**Treatment**	**Wet weight (gr)**	**Dry weight (gr)**	**Diameter (mm)**	**Gonadosomatic Index**	**Specific growth rate (SGR)**
Control (T0)	272.4±16.85	n.d.	85.16±2.46	n.d.	
Gracilaria	317.36±13.45	51.82±4.05	91.62±1.28	22.36±2.43	0.275±0.063
Pellet	291.97±26.52	48.18±3.25	87.77±2.15	23.00±2.48	0.104±0.074
Ulva	329.04±20.5	52.81±3.67	86.77±1.58	26.70±2.59	0.334±0.075

Table S3. OTU count and number of reads for each of the examined samples from a particular gut region and diet regime (n=54).

Diet	Sampling region	Sample ID	Total number of observation	OTUs count
Gracilaria	Esophagus	Matan73.G9E3	19753	216
		Matan58.G2E3	4414	78
		Matan46.G9E2	19287	201
		Matan43.G7E2	15768	153
		Matan31.G2E2	18080	134
		Matan19.G9E1	35272	137
		Matan16.G7E1	16865	140
	Stomach	Matan74.G9S3	25978	143
		Matan71.G7S3	20249	122
		Matan47.G9S2	17407	123
		Matan44.G7S2	21338	179
		Matan32.G2S2	18853	88
		Matan20.G9S1	14087	110
		Matan17.G7S1	15568	100
	Intestine	Matan75.G9I3	18825	39
		Matan72.G7I3	21228	127
		Matan48.G9I2	17421	168
		Matan45.G7I2	14472	179
		Matan33.G2I2	23420	113
		Matan21.G9I1	20152	79
		Matan18.G7I1	18221	85
Ulva	Esophagus	Matan79.U11E3	16188	161
		Matan67.U6E3	18179	91
		Matan55.U1E3	17252	118
		Matan40.U6E2	18227	91
		Matan1.U1E1	6040	61
	Stomach	Matan80.U11S3	19628	215
		Matan68.U6S3	20801	123
		Matan56.U1S3	23401	114
		Matan41.U6S2	17552	78
		Matan26.U11S1	16602	113
	Intestine	Matan81.U11I3	19967	188
		Matan42.U6I2	18187	126
		Matan30.U1I2	16034	118
		Matan27.U11I1	34871	158
		Matan15.U6I1	17014	132
Pellet	Esophagus	Matan22.P10E1	14766	67
		Matan37.P5E2	18069	171
		Matan49.P10E2	16747	101
		Matan61.P3E3	18017	175
		Matan64.P5E3	20299	132
		Matan76.P10E3	12028	109
	Stomach	Matan35.P3S2	14948	91
		Matan38.P5S2	18641	47
		Matan62.P3S3	14122	61
		Matan65.P5S3	20249	36
		Matan77.P10S3	20677	128
		Matan8.P3S1	27076	29
	Intestine	Matan24.P10I1	19998	145
		Matan36.P3I2	17728	146
		Matan39.P5I2	13475	49
		Matan63.P3I3	8195	12
		Matan78.P10I3	22934	147
		Matan9.P3I1	13972	66

Table S4. OTU count for the various identified phyla in sea urchin gut samples (n=54).

Phyla	OTUs count
Proteobacteria	122
Bacteroidetes	111
Unassigned	60
Firmicutes	51
Planctomycetes	29
Spirochaetes	14
Acidobacteria	11
Tenericutes	9
Cyanobacteria	8
Verrucomicrobia	5
Chloroflexi	3
Dadabacteria	2
Epsilonbacteraeota	2
Fusobacteria	2
Lentisphaerae	2
Cloacimonetes	1
Thaumarchaeota	1
Thermotogae	1

Table S5. Shared edges between GMA association networks in different diets or gut regions (n=54) indicating type of relationship (co-occurrence or co-exclusion) and the participating nodes.

**Examined variable (diet or gut region)**	**OTU-1**	**OTU-2**	**Type of relationship co-occurrence (+) or co-exclusion (-)**	**OTU-1 Taxonomic identification**	**OTU-2 Taxonomic identification**
Diet	denovo28887	denovo10071	-	Unassigned	*Bacteria; Bacteroidetes; Bacteroidia; Bacteroidales; vadinHA21;* uncultured Bacteroidetes bacterium
	denovo34968	denovo29071	-	*Bacteria; Firmicutes; Clostridia; Clostridiales; Caldicoprobacteraceae; Caldicoprobacter;* uncultured bacterium	*Bacteria; Cyanobacteria; Melainabacteria; Gastranaerophilales*
	denovo40181	denovo29071	-	*Bacteria; Bacteroidetes; Bacteroidia; Bacteroidales; Prolixibacteraceae; Roseimarinus;* uncultured Bacteroidetes bacterium	*Bacteria; Cyanobacteria; Melainabacteria; Gastranaerophilales*
	denovo6275	denovo39613	-	*Bacteria; Cyanobacteria; Melainabacteria; Gastranaerophilales*	Unassigned
Gut Region	denovo12912	denovo10071	+	*Bacteria; Proteobacteria; Gammaproteobacteria; Vibrionales; Vibrionaceae; Vibrio*	*Bacteria; Bacteroidetes; Bacteroidia; Bacteroidales; vadinHA21; uncultured Bacteroidetes bacterium*

	denovo12912	denovo31035	-	*Bacteria; Proteobacteria; Gammaproteobacteria; Vibrionales; Vibrionaceae; Vibrio*	*Bacteria; Tenericutes; Mollicutes; Entomoplasmatales; Entomoplasmatales Incertae Sedis; Candidatus Hepatoplasma*
	denovo12912	denovo7299	-	*Bacteria; Proteobacteria; Gammaproteobacteria; Vibrionales; Vibrionaceae; Vibrio*	*Bacteria; Bacteroidetes; Bacteroidia; Bacteroidetes VC2.1 Bac22;* uncultured bacterium
	denovo28887	denovo14833	-	Unassigned	Unassigned
	denovo28887	denovo23305	-	Unassigned	*Bacteria; Spirochaetes; V2072-189E03;* uncultured organism
	denovo37899	denovo22419	-	*Bacteria; Spirochaetes; Spirochaetia; Spirochaetales; Spirochaetaceae; Spirochaeta 2*	*Bacteria; Bacteroidetes; Bacteroidia; Bacteroidales; Marinifilaceae;* uncultured; uncultured Bacteroidetes bacterium
	denovo39075	denovo23305	+	Unassigned	*Bacteria; Spirochaetes; V2072-189E03;* uncultured organism
	denovo6275	denovo23305	-	*Bacteria; Cyanobacteria; Melainabacteria; Gastranaerophilales*	*Bacteria; Spirochaetes; V2072-189E03;* uncultured organism
	denovo34968	denovo29071	-	*Bacteria; Firmicutes; Clostridia; Clostridiales; Caldicoprobacteraceae; Caldicoprobacter;* uncultured bacterium	*Bacteria; Cyanobacteria; Melainabacteria; Gastranaerophilales*
	denovo7299	denovo31035	-	*Bacteria; Bacteroidetes; Bacteroidia; Bacteroidetes VC2.1 Bac22;* uncultured bacterium	*Bacteria; Tenericutes; Mollicutes; Entomoplasmatales; Entomoplasmatales Incertae Sedis; Candidatus Hepatoplasma*
	denovo7539	denovo31035	-	*Bacteria; Bacteroidetes; Bacteroidia; Bacteroidales; vadinHA21;* uncultured Bacteroidetes bacterium	*Bacteria; Tenericutes; Mollicutes; Entomoplasmatales; Entomoplasmatales Incertae Sedis; Candidatus Hepatoplasma*
	denovo7539	denovo3738	-	*Bacteria; Bacteroidetes; Bacteroidia; Bacteroidales; vadinHA21;* uncultured Bacteroidetes bacterium	*Bacteria; Firmicutes; Clostridia; Clostridiales; Family XII; Fusibacter; uncultured Fusibacter sp.*
	denovo6275	denovo39613	-	*Bacteria; Cyanobacteria; Melainabacteria; Gastranaerophilales*	Unassigned
	denovo7539	denovo7299	-	*Bacteria; Bacteroidetes; Bacteroidia; Bacteroidales; vadinHA21;* uncultured Bacteroidetes bacterium	*Bacteria; Bacteroidetes; Bacteroidia; Bacteroidetes VC2.1 Bac22;* uncultured bacterium

Table S6. Topology properties of hub microbes in GMA association networks presenting number of degrees, closeness, and betweenness indices in the examined network of the different diets or gut regions (n=54).

**Network**	**OTU ID**	**No. of Degrees**	**Closeness**	**Betweenness**	**Taxonomic identification**
General	denovo31035	5	0.068783	0.109005	*Bacteria; Tenericutes; Mollicutes; Entomoplasmatales; Entomoplasmatales Incertae Sedis; Candidatus Hepatoplasma*
	denovo3738	5	0.068783	0.07109	*Bacteria; Firmicutes; Clostridia; Clostridiales; Family XII; Fusibacter;* uncultured Fusibacter sp.
Gracilaria	denovo31035	9	0.183333	0.073811	*Bacteria; Tenericutes; Mollicutes; Entomoplasmatales; Entomoplasmatales Incertae Sedis; Candidatus Hepatoplasma*
	denovo15828	7	0.176471	0.012543	*Bacteria; Bacteroidetes; Bacteroidia; Bacteroidetes VC2.1 Bac22;* uncultured Bacteroidetes bacterium
Pellet	denovo10071	6	0.206061	0.041413	*Bacteria; Bacteroidetes; Bacteroidia; Bacteroidales; vadinHA21;* uncultured Bacteroidetes bacterium
	denovo28887	6	0.206061	0.041413	Unassigned
Ulva	denovo13167	17	0.112643	0.557377	Unassigned
	denovo23305	18	0.141768	0.566667	*Bacteria; Spirochaetes; V2072-189E03;* uncultured organism
	denovo29071	17	0.112643	0.557377	*Bacteria; Cyanobacteria; Melainabacteria; Gastranaerophilales*
	denovo34968	17	0.112643	0.557377	*Bacteria; Firmicutes; Clostridia; Clostridiales; Caldicoprobacteraceae; Caldicoprobacter;* uncultured bacterium
	denovo6275	17	0.112643	0.557377	*Bacteria; Cyanobacteria; Melainabacteria; Gastranaerophilales*
Esophagus	denovo10071	6	0.414634	0.057692	*Bacteria; Bacteroidetes; Bacteroidia; Bacteroidales; vadinHA21;* uncultured Bacteroidetes bacterium
	denovo3738	7	0.4	0.017768	*Bacteria; Firmicutes; Clostridia; Clostridiales; Family XII; Fusibacter;* uncultured Fusibacter sp.
	denovo6275	6	0.425	0.050391	*Bacteria; Cyanobacteria; Melainabacteria; Gastranaerophilales*
Stomach	denovo37437	5	0.390805	0.064583	*Bacteria; Bacteroidetes; Bacteroidia; Bacteroidales; Marinifilaceae;* uncultured; uncultured Bacteroidetes bacterium
Intestine	denovo6275	6	0.419753	0.055468	*Bacteria; Cyanobacteria; Melainabacteria; Gastranaerophilales*
	denovo23618	7	0.441558	0.10324	*Bacteria; Proteobacteria; Gammaproteobacteria; Vibrionales; Vibrionaceae; Photobacterium;* uncultured gamma proteobacterium

Table S7. Topology properties of identified driver nodes in GMA association networks presenting Jaccard Edge Index, NESH score and delta betweenness centrality (normalized to the average path length) indices as measured in pairwise analyses of control versus case networks. Real drivers, i.e. nodes that were identified as driver in a specific niche against each of two other niches, are indicated in bold.

**Compared networks pair (case vs. control)**	**Drivers**	**OTU**	**Jaccard - score**	**NESH-score**	**DelBet (Δ betweenness)**	**Taxonomy (SILVA)**
Case network: *Gracilaria* vs. Control network: Pellets	Control drivers	denovo3738	0.083	2.485	-0.132	*Bacteria; Firmicutes; Clostridia; Clostridiales; Family XII; Fusibacter; uncultured Fusibacter sp.*
		denovo15828	0	2.414	-0.022	*Bacteria; Bacteroidetes; Bacteroidia; Bacteroidetes VC2.1 Bac22;* uncultured Bacteroidetes bacterium
		denovo39075	0.111	2.303	-0.089	Unassigned
		denovo23305	0.125	2.256	-0.294	*Bacteria; Spirochaetes; V2072-189E03; uncultured organism*
		denovo32222	0	2.08	-0.01	*Bacteria; Bacteroidetes; Bacteroidia; Bacteroidales; Prolixibacteraceae; Roseimarinus;* uncultured Bacteroidetes bacterium
		denovo36317		2.01	-0.505	*Bacteria; Proteobacteria; Deltaproteobacteria; Desulfobacterales; Desulfobulbaceae; Desulfotalea*
		denovo22419	0	2.007	0.037	*Bacteria; Bacteroidetes; Bacteroidia; Bacteroidales; Marinifilaceae; uncultured;* uncultured Bacteroidetes bacterium
		**denovo36058**	**0.1**	**1.855**	**-0.341**	***Bacteria; Bacteroidetes; Bacteroidia; Bacteroidales; Prolixibacteraceae; Roseimarinus;* uncultured Bacteroidetes bacterium**
	Case drivers	denovo7539	0	2.718	0.174	*Bacteria; Bacteroidetes; Bacteroidia; Bacteroidales; vadinHA21;* uncultured Bacteroidetes bacterium
		**denovo22514**	0	2.511	0.236	*Bacteria; Spirochaetes; Spirochaetia; Spirochaetales; Spirochaetaceae; Spirochaeta 2*
		denovo7299	0	2.295	0.147	*Bacteria; Bacteroidetes; Bacteroidia; Bacteroidetes VC2.1 Bac22;* uncultured bacterium
		**denovo28887**	**0.133**	**2.285**	**0.37**	**Unassigned**
		denovo23618	0	2.164	0.012	*Bacteria; Proteobacteria; Gammaproteobacteria; Vibrionales; Vibrionaceae; Photobacterium;* uncultured gamma proteobacterium
		denovo37437	0	2.03	0.916	*Bacteria; Bacteroidetes; Bacteroidia; Bacteroidales; Marinifilaceae; uncultured;* uncultured Bacteroidetes bacterium

		denovo12912	0	1.935	0.369	*Bacteria; Proteobacteria; Gammaproteobacteria; Vibrionales; Vibrionaceae; Vibrio*
		denovo23690	0	1.873	0.47	*Bacteria; Spirochaetes; Spirochaetia; Spirochaetales; Spirochaetaceae; Sediminispirochaeta;* uncultured bacterium
		denovo14715	0	1.848	0.021	*Bacteria; Spirochaetes; Spirochaetia; Spirochaetales; Spirochaetaceae; Sediminispirochaeta;* uncultured bacterium
Case network: *Gracilaria* vs. Control network: *Ulva*	Control drivers	denovo39075	0.125	1.795	-0.876	Unassigned
		denovo26431	0	1.727	-0.109	*Bacteria; Firmicutes; Clostridia; Clostridiales; Lachnospiraceae;* uncultured; uncultured bacterium
		**denovo31035**	**0.167**	**1.712**	**-0.25**	***Bacteria; Tenericutes; Mollicutes; Entomoplasmatales; Entomoplasmatales Incertae Sedis; Candidatus Hepatoplasma***
	Case drivers	**denovo28887**	**0.083**	**2.659**	**0.421**	**Unassigned**
		denovo3738	0.176	1.872	0.463	*Bacteria; Firmicutes; Clostridia; Clostridiales; Family XII; Fusibacter;* uncultured Fusibacter sp.
		denovo10071	0.25	1.83	0.761	*Bacteria; Bacteroidetes; Bacteroidia; Bacteroidales; vadinHA21;* uncultured Bacteroidetes bacterium
		**denovo22514**	**0.048**	**1.784**	**0.224**	***Bacteria; Spirochaetes; Spirochaetia; Spirochaetales; Spirochaetaceae; Spirochaeta 2***
		denovo31563	0	1.701	0.049	*Bacteria; Spirochaetes; Spirochaetia; Spirochaetales; Spirochaetaceae; Spirochaeta 2;* uncultured bacterium
Case network: *Ulva* vs. Control network: Pellet	Control drivers	denovo12806	0	1.944	-0.257	*Bacteria; Fusobacteria; Fusobacteriia; Fusobacteriales; Fusobacteriaceae; Propionigenium;* uncultured bacterium
		**denovo31035**	**0.059**	**1.86**	**-0.184**	***Bacteria; Tenericutes; Mollicutes; Entomoplasmatales; Entomoplasmatales Incertae Sedis; Candidatus Hepatoplasma***
		denovo13167	0.045	1.807	-0.039	Unassigned
	Case drivers	denovo26431	0	2.375	0.057	*Bacteria; Firmicutes; Clostridia; Clostridiales; Lachnospiraceae; uncultured;* uncultured bacterium
		denovo31563	0	2.35	0.439	*Bacteria; Spirochaetes; Spirochaetia; Spirochaetales; Spirochaetaceae; Spirochaeta 2;* uncultured bacterium
		denovo28887	0.143	2.196	0.041	Unassigned
		denovo10071	0.125	2.125	0.034	*Bacteria; Bacteroidetes; Bacteroidia; Bacteroidales; vadinHA21;* uncultured Bacteroidetes bacterium
		**denovo36058**	**0**	**2.125**	**0.619**	***Bacteria; Bacteroidetes; Bacteroidia; Bacteroidales; Prolixibacteraceae;***

						Roseimarinus; **uncultured Bacteroidetes bacterium**
		denovo37899	0.067	1.892	0.299	*Bacteria; Spirochaetes; Spirochaetia; Spirochaetales; Spirochaetaceae; Spirochaeta 2*
		denovo9583	0	1.852	0.534	*Bacteria; Bacteroidetes; Bacteroidia; Flavobacteriales; Cryomorphaceae; NS10 marine group;* uncultured Bacteroidetes bacterium
Case network: Esophagus vs. Control network: Stomach	Control drivers	denovo14715	0.062	2.062	-0.092	*Bacteria; Spirochaetes; Spirochaetia; Spirochaetales; Spirochaetaceae; Sediminispirochaeta;* uncultured bacterium
		denovo39075	0.15	1.975	-0.099	Unassigned
		denovo23690	0	1.875	-0.061	*Bacteria; Spirochaetes; Spirochaetia; Spirochaetales; Spirochaetaceae; Sediminispirochaeta;* uncultured bacterium
	Case drivers	denovo22514	0	2.292	0.773	*Bacteria; Spirochaetes; Spirochaetia; Spirochaetales; Spirochaetaceae; Spirochaeta 2*
		denovo3738	0.067	2.225	0.171	*Bacteria; Firmicutes; Clostridia; Clostridiales; Family XII; Fusibacter;* uncultured Fusibacter sp.
		**denovo6214**	**0.263**	**1.888**	**0.559**	***Bacteria; Bacteroidetes; Bacteroidia; Bacteroidales; Prolixibacteraceae; Roseimarinus***
		denovo39613	0.222	1.84	0.051	*Unassigned*
Case network: Esophagus vs. Control network: Intestine	Control drivers	denovo12806	0.118	1.974	-0.017	*Bacteria; Fusobacteria; Fusobacteriia; Fusobacteriales; Fusobacteriaceae; Propionigenium;* uncultured bacterium
		**denovo14715**	**0.133**	**1.9**	**-0.268**	***Bacteria; Spirochaetes; Spirochaetia; Spirochaetales; Spirochaetaceae; Sediminispirochaeta;*** **uncultured bacterium**
	Case drivers	**denovo6214**	**0.15**	**2.2**	**0.69**	***Bacteria; Bacteroidetes; Bacteroidia; Bacteroidales; Prolixibacteraceae; Roseimarinus***
		denovo9583	0.167	2.132	0.153	*Bacteria; Bacteroidetes; Bacteroidia; Flavobacteriales; Cryomorphaceae; NS10 marine group*; uncultured Bacteroidetes bacterium
		denovo37437	0.1	1.875	0.204	*Bacteria; Bacteroidetes; Bacteroidia; Bacteroidales; Marinifilaceae*; uncultured; uncultured Bacteroidetes bacterium
		denovo7856	0.1	1.875	0.119	Unassigned
Case network: Stomach vs. Control network: Intestine	Control drivers	denovo40181	0	2.042	-0.455	*Bacteria; Bacteroidetes; Bacteroidia; Bacteroidales; Prolixibacteraceae; Roseimarinus;* uncultured Bacteroidetes bacterium
		**denovo14715**	**0**	**2**	**-0.176**	***Bacteria; Spirochaetes; Spirochaetia; Spirochaetales; Spirochaetaceae; Sediminispirochaeta;*** **uncultured bacterium**

		denovo15125	0.077	1.813	-0.123	*Bacteria; Bacteroidetes; Bacteroidia; Bacteroidales; Marinifilaceae;* uncultured; uncultured Bacteroidetes bacterium
	Case drivers	denovo37437	0.083	2.155	0.154	*Bacteria; Bacteroidetes; Bacteroidia; Bacteroidales; Marinifilaceae;* uncultured; uncultured Bacteroidetes bacterium
		denovo7856	0	2.136	0.106	Unassigned
		denovo6214	0.062	2.009	0.131	*Bacteria; Bacteroidetes; Bacteroidia; Bacteroidales; Prolixibacteraceae; Roseimarinus*
		denovo9583	0.312	1.893	0.473	*Bacteria; Bacteroidetes; Bacteroidia; Flavobacteriales; Cryomorphaceae; NS10 marine group;* uncultured Bacteroidetes bacterium

Table S8. BLAST (Basic Local Alignment Search Tool) results of the closest phylogenetic species that was identified for each of the OTUs that were identified in current research as core, core-generalist, generalist, specialist, unique, or hub microbes. The bio project number in the NCBI database, where the genome of any of the assigned species is available, is provided. Not found = no genome was available for the closest phylogenetically-related species.

Definition	OTU ID	Species	Bio Project
Core	denovo32222	*Roseimarinus sediminis DSM 28824*	PRJNA546787
	denovo7299	*Bacteroidia*	Not found
	denovo426	*Ruegeria pomeroyi DSS-3*	PRJNA281
	denovo12912	*Vibrio parahaemolyticus RIMD 2210633*	PRJNA360
Core generalist	denovo7539	*uncultured Marinilabiliaceae bacterium*	PRJEB34458
	denovo3738	*Fusibacter tunisiensis*	PRJNA695606
	denovo31035	*Candidatus Hepatoplasma crinochetorum Av*	PRJNA229465
Generalist	denovo34858	Unassigned	
	denovo2269	Unassigned	
	denovo36881	*Celeribacter halophilus*	PRJNA262270
	denovo582	Unassigned	
	denovo20261	Unassigned	
	denovo29824	Unassigned	

	denovo34968	*Caldicoprobacter faecalis*	PRJEB17439
	denovo2328	*Achromobacter xylosoxidans*	PRJNA362984
	denovo23618	*Photobacterium leiognathi subsp. mandapamensis svers.1.1.*	PRJDA64795
	denovo29071	*Gloeobacter kilaueensis*	PRJNA162637
	denovo20151	*Mariniblastus fucicola*	PRJNA485700
	denovo33543	*Planctomyces sp.*	PRJNA592128
	denovo18322	*Methyloceanibacter caenitepidi*	PRJDB3104
	denovo36317	*Desulfotalea psychrophila LSv54*	PRJNA12751
	denovo11613	*Rubripirellula amarantea*	PRJNA485700
Specialist	denovo2310	*Ulvibacter antarcticus*	PRJNA363566
	denovo43468	*Reichenbachiella agariperforans*	PRJEB18330
	denovo13979	*Roseivirga ehrenbergii*	PRJNA519310
	denovo32773	Unassigned	
	denovo1069	*Candidatus Nitrosopumilus*	Not found
	denovo9503	*Acidiluteibacter ferrifornacis*	PRJNA595662
	denovo10645	*Lentimicrobiaceae*	Not found
	denovo3573	*Veillonella parvula*	PRJEB36442
	denovo6928	*Phyllobacterium myrsinacearum*	PRJNA520296
	denovo38262	*Urechidicola croceus*	PRJNA341557
	denovo5098	*Lentimicrobiaceae*	Not found
	denovo9355	*Desulfobacter hydrogenophilus*	PRJNA523089
Ulva unique	denovo10328	*Roseimarinus sediminis DSM 28824*	PRJNA546787
	denovo43431	*Roseimarinus sediminis DSM 28824*	PRJNA546787
	denovo9352	*Roseimarinus sediminis DSM 28824*	PRJNA546787
	denovo4973	*Roseimarinus sediminis DSM 28824*	PRJNA546787
	denovo36133	uncultured Marinilabiliaceae bacterium	PRJEB34458
	denovo6275	*Gloeobacter kilaueensis*	PRJNA162637
	denovo25524	*Natranaerovirga pectinivora*	PRJNA500302
	denovo19840	*Spirochaeta isovalerica*	PRJNA632313
	denovo23305	*Spirochaetes*	Not found
	denovo11880	*Mollicutes*	Not found
	denovo30083	Unassigned	
	denovo12333	Unassigned	
	denovo10114	Unassigned	
HUB			
Gracilaria unique	denovo26795	Planctomycetes	Not found
	denovo796	*Persicobacter sp. CCB-QB2*	PRJNA263536

Gracilaria and *Ulva* unique	denovo43602	Unassigned	
General	denovo31035	*Candidatus Hepatoplasma crinochetorum Av*	PRJNA229465
	denovo3738	*Fusibacter tunisiensis*	PRJNA695606
Gracilaria	denovo31035	*Candidatus Hepatoplasma crinochetorum Av*	PRJNA229465
	denovo15828	*Lentimicrobium saccharophilum*	PRJDB4040
Pellet	denovo10071	*Bacteroidales*	Not found
	denovo28887	Unassigned	
Ulva	denovo13167	Unassigned	
	denovo23305	*Spirochaetes*	Not found
	denovo29071	*Gloeobacter kilaueensis*	PRJNA162637
	denovo34968	*Caldicoprobacter faecalis*	PRJEB17439
	denovo6275	*Gloeobacter kilaueensis*	PRJNA162637
Esophagus	denovo10071	*Bacteroidales*	Not found
	denovo3738	*Fusibacter tunisiensis*	PRJNA695606
	denovo6275	*Gloeobacter kilaueensis*	PRJNA162637
Stomach	denovo37437	*Sunxiuqinia elliptica*	PRJEB17263
Intestine	denovo6275	*Gloeobacter kilaueensis*	PRJNA162637
	denovo23618	*Photobacterium leiognathi subsp. mandapamensis svers.1.1.*	PRJDA64795
